# Hippocampal spatial view cells for memory and navigation, and their underlying connectivity in humans

**DOI:** 10.1002/hipo.23467

**Published:** 2022-09-07

**Authors:** Edmund T. Rolls

**Affiliations:** ^1^ Oxford Centre for Computational Neuroscience Oxford UK; ^2^ Department of Computer Science University of Warwick Coventry UK

**Keywords:** concept cells in humans and macaques, episodic memory, hippocampus, navigation, parahippocampal scene area, place cells, spatial view cells

## Abstract

Hippocampal and parahippocampal gyrus spatial view neurons in primates respond to the spatial location being looked at. The representation is allocentric, in that the responses are to locations “out there” in the world, and are relatively invariant with respect to retinal position, eye position, head direction, and the place where the individual is located. The underlying connectivity in humans is from ventromedial visual cortical regions to the parahippocampal scene area, leading to the theory that spatial view cells are formed by combinations of overlapping feature inputs self‐organized based on their closeness in space. Thus, although spatial view cells represent “where” for episodic memory and navigation, they are formed by ventral visual stream feature inputs in the parahippocampal gyrus in what is the parahippocampal scene area. A second “where” driver of spatial view cells are parietal inputs, which it is proposed provide the idiothetic update for spatial view cells, used for memory recall and navigation when the spatial view details are obscured. Inferior temporal object “what” inputs and orbitofrontal cortex reward inputs connect to the human hippocampal system, and in macaques can be associated in the hippocampus with spatial view cell “where” representations to implement episodic memory. Hippocampal spatial view cells also provide a basis for navigation to a series of viewed landmarks, with the orbitofrontal cortex reward inputs to the hippocampus providing the goals for navigation, which can then be implemented by hippocampal connectivity in humans to parietal cortex regions involved in visuomotor actions in space. The presence of foveate vision and the highly developed temporal lobe for object and scene processing in primates including humans provide a basis for hippocampal spatial view cells to be key to understanding episodic memory in the primate and human hippocampus, and the roles of this system in primate including human navigation.

## 1 INTRODUCTION

The aims of this article are to describe the properties of the spatial view cells found in primates including humans; to consider evidence on their roles in memory and navigation; to compare them to place cells in rodents and to address the computational bases for the similarities and differences; to consider recent evidence on the connectivity of the hippocampus in humans that helps to elucidate the different sets of inputs to spatial view cells including visual scene, idiothetic, and reward information; and to consider how information about spatial view and about objects can be recalled from the hippocampus back to the neocortex during episodic memory retrieval. It is argued that hippocampal spatial view cells are fundamental to understanding episodic memory in humans, and important in navigational strategies in humans.

This article in the Special Issue of Hippocampus (2023) entitled “Hippocampal system neurons encoding views in different species,” has the aim of describing the evidence about the key discoveries and properties of spatial view cells in primates, and then developing our understanding of how this system operates in primates including humans by showing, based on recent evidence, how the hippocampal system is connected to its inputs and outputs in humans. This is in the context that the connectivity of each brain region is a key component in developing an understanding of what is computed in each brain region, and how it is computed (Rolls, 2021a). Indeed, to understand brain computations, it is important to consider evidence about what is represented in a brain region from neuronal recordings and brain activations, the connectivity of the brain region with other brain regions, the internal neuronal network connectivity of a brain region, the effects of damage to a brain region, and so forth (Rolls, 2021a), and this article considers some of the key discoveries and evidence of these types that are important for understanding how the hippocampus operates in primates including humans. This article builds on material presented by Rolls and Wirth (2018), which is included here so that this article in this Special Issue of Hippocampus provides a comprehensive overview, but adds much new evidence and new concepts from recent human connectivity and computational studies with Figures 5, 6, 7, 8, 9, 10, 11, 12 referring to these new investigations (Huang et al., 2021; Ma et al., 2022; Rolls, 2020; Rolls, 2021a; Rolls, 2021b; Rolls, 2022; Rolls, Deco, et al., 2022b; Rolls, Deco, et al., 2022c; Rolls, Deco, et al., 2022d; Rolls, Deco, et al., 2022e; Rolls & Mills, 2019), as well as recent evidence from sources.

Spatial view cells are found in the primate hippocampus and parahippocampal gyrus (Georges‐François et al., 1999; Robertson et al., 1998; Rolls et al., 1997b; Rolls et al., 1998; Rolls et al., 2005; Rolls & Xiang, 2005). In humans, the parahippocampal place area (better called the parahippocampal scene area [PSA] as it responds to viewed scenes not the place where the individual is located) (Epstein, 2005; Epstein, 2008; Epstein & Baker, 2019; Epstein & Julian, 2013; Epstein & Kanwisher, 1998; Kamps et al., 2016; Natu et al., 2021; Sulpizio et al., 2020) is found in the posterior part of the parahippocampal gyrus and extends into the ventromedial visual areas VMV1‐3 (Sulpizio et al., 2020). It is proposed that spatial view cells are the type of neuron found in the human PSA, and that this is a route via which hippocampal spatial view cells receive their information about and selectivity for locations in scenes (Rolls, Deco, et al., 2022b; Rolls, Deco, et al., 2022c; Rolls, Wirth, et al., 2022).

## 2 BACKGROUND

Lesion studies in nonhuman primates have shown that hippocampal damage (or damage to the fornix) leads to learning deficits about locations “out there” in space where objects are located, and about the locations out there in space where responses are required (Gaffan, 1994; Murray et al., 2017; Parkinson et al., 1988), and there is corresponding evidence for humans (Crane & Milner, 2005; Smith & Milner, 1981). In macaques, parahippocampal cortex damage even impairs object‐location associations with just one pair of trial‐unique stimuli to be remembered (Malkova & Mishkin, 2003). Further, neurotoxic lesions of the primate hippocampus impair spatial scene memory (Murray et al., 1998). Monkeys with fornix section also are impaired to use a viewed spatial location to learn which object to choose (Gaffan & Harrison, 1989). Hippocampal damage in macaques impairs the ability to remember the locations in an open field of rewarded objects (Hampton et al., 2004). Also, in a foraging task, monkeys with hippocampal lesions could not use allocentric, room‐based, spatial cues to find food (Banta Lavenex & Lavenex, 2009). Thus, lesion evidence implicates the primate hippocampus in the memory of locations “out there” in space, and spatial scene memory.

In contrast, in rodents, the emphasis has been on the representation in the hippocampus of the place where the rodent is located, as shown by recordings from hippocampal place cells (Hartley et al., 2014; Markus et al., 1995; McNaughton et al., 1983; Muller et al., 1991; O'Keefe, 1979; O'Keefe, 1984) and entorhinal cortex cells representing a grid of places where the rodent is located (Edvardsen et al., 2020; Kropff & Treves, 2008; Moser et al., 2014; Moser et al., 2015).

## 3 ON THE NATURE OF SPATIAL VIEW REPRESENTATIONS IN PRIMATES

Given the evidence from the effects of lesions in primates and the evidence for spatial representations in rodents (see McNaughton et al., 1983; Morris et al., 1982; Muller et al., 1991; O'Keefe, 1984), Rolls et al. investigated the nature of spatial representations in macaques, and how hippocampal neuronal activity might be related to memory tasks including object‐location and reward‐location memory.

### 3.1 The discovery of primate spatial view cells and place cells

First, we tested whether neurons responded to different locations “out there” in space in an object‐location memory task. The monkey had to remember where on a video screen a particular visual stimulus had been seen previously. We discovered that some primate hippocampal neurons responded differently to different locations in space “out there” on the screen, and some neurons combined this with what picture had been shown in that location previously (Cahusac et al., 1989; Rolls et al., 1989).

Next, we tested for spatial view versus place‐related representations of primate hippocampal and parahippocampal gyrus neurons in a spatial environment. Macaques were moved on a platform mounted on a free‐moving robot in an open laboratory or on wheels in a cue‐controlled 2 m × 2 m × 2 m spatial environment. In the cue‐controlled environment, there was one room cue on each of the four walls, and the room cues could be moved. The test conditions allowed factors that might account for spatial firing of the hippocampal neurons, including the spatial location where the monkey looked, the place where the monkey was, and the head direction of the monkey, to be analyzed (Rolls & O'Mara, 1995). We discovered that the majority of the neurons with spatial responses had spatial view responses, that depended on where the monkey was looking in the environment, but not on the place of the monkey in the environment or on head direction (Rolls & O'Mara, 1995).

In addition, some neurons with place‐related firing in the primate were discovered: some neurons responded, for example, to the place where the macaque was located, to movement to a place, or to spatial view depending on the place where the monkey was located (Rolls & O'Mara, 1995).

### 3.2 An allocentric representation of space in the primate hippocampus

It is important to define the reference frames for spatial representations, as they are relevant to understanding the functions that can be performed. An egocentric frame of reference (relative to the head or body) is useful for actions made in nearby space. An allocentric frame of reference (i.e., world‐based coordinates) is useful for remembering the location of objects and rewards in the world, independently of the one's body or head orientation or eye position. In primates and other animals with good vision or with echolocation, this can be independent of the place where one is located, though in rodents, the allocentric representation is more likely to be of the place where one is located, as distance vision is poor, and reliance is instead on local somatosensory cues using the vibrissae, local odors, and so forth. The discovery that some hippocampal neurons respond to the location on a video screen in front of the macaque (Rolls et al., 1989), and can even reflect the object shown in a particular location on the screen (Cahusac et al., 1989), raised the issue of which coordinate frame is used by the primate hippocampus. Feigenbaum and Rolls (1991) analyzed whether these spatial view neurons utilize allocentric or egocentric spatial coordinates. They moved the video screen and the macaque relative to each other, and to different places in the room. Then, 46% of the spatial neurons had firing that occurred to the same spatial location viewed on the display, or viewed in the laboratory, when the macaque was rotated or moved to a different place in the room. Thus, these hippocampal cells had spatial representations in allocentric (i.e., world‐based) and not in egocentric (relative to the body or head) coordinates. Also, 10% of the hippocampal spatial neurons had firing that stayed in the same location relative to the monkey's head/body axis when the video monitor was displaced, or the macaque was rotated, or was displaced to a different place in the room. Thus, 10% of the neurons represented space in egocentric coordinates, that is, relative to the head.

Feigenbaum and Rolls (1991) in addition showed that there were two types of allocentric encoding. For the majority of the neurons, the spatial field was in terms of its location on the video screen, independently of the place of the screen relative to the monkey's head axis, and independently of the place of the macaque and the monitor in the room. This type of neuron was termed “local frame of reference” allocentric, in that spatial fields of these neurons were defined by the local spatial frame that was provided by the video monitor. For the second type of allocentric encoding, the spatial field was defined by the location in the laboratory toward which the monkey was fixating, and was relatively independent of position with respect to the monkey's body/head axis or to position on the face of the video screen. This type of neuron was termed “absolute” allocentric, in that their spatial view fields were defined by the location in the laboratory that the animal foveated (Feigenbaum & Rolls, 1991).

Allocentric encoding is also a property of rodent hippocampal place cells, but the encoding is of the place where the rodent (rat or mouse) is, not of where in space the rodent is looking. However, the parallel is that in both cases allocentric encoding is found, and this allocentric representation is important for hippocampal computation and potentially for remembering where objects have been found in the environment (Kesner & Rolls, 2015; Rolls, 2018a; Rolls, 2021a; Rolls, 2021b; Rolls & Wirth, 2018).

### 3.3 Responses of hippocampal and parahippocampal gyrus allocentric spatial view neurons during active locomotion

**FIGURE 1:**
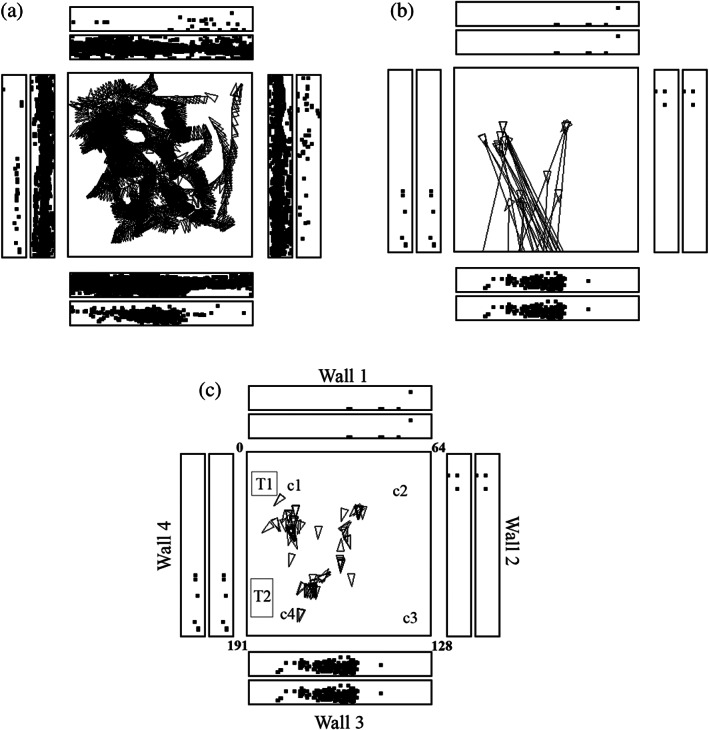
A hippocampal spatial view cell (az033) recorded while a monkey walked around in an open field area 2.5 × 2.5 m shown as the square within a rich and large laboratory environment. In (a), every time that the cells fired is shown by a spot in the outer rectangles each of which represents one of the four walls of the room. The inner rectangles show where the monkey looked on the walls. The neurons have a spatial view field on wall 3. The places to which the monkey walked are shown by the triangles, with the pointed end showing the head direction. (b) Shows some of the many different places at which the monkey was located when the neuron fired, and the lines show where the monkey was fixating when the spatial view cell fired. (c) Provides more evidence about the places where the monkey was located when the cell fired because he was looking at the view field on wall 3. This helps to show that the neuron responds to spatial view, and not to the place where the monkey was located. C1 to c4 are cups containing food to encourage the monkey to forage. T1 was a trolley and T2 a table. Details are provided by Georges‐François et al. (1999). Videos to illustrate the firing of spatial view neurons are described in the Data Availability Statement

**FIGURE 2:**
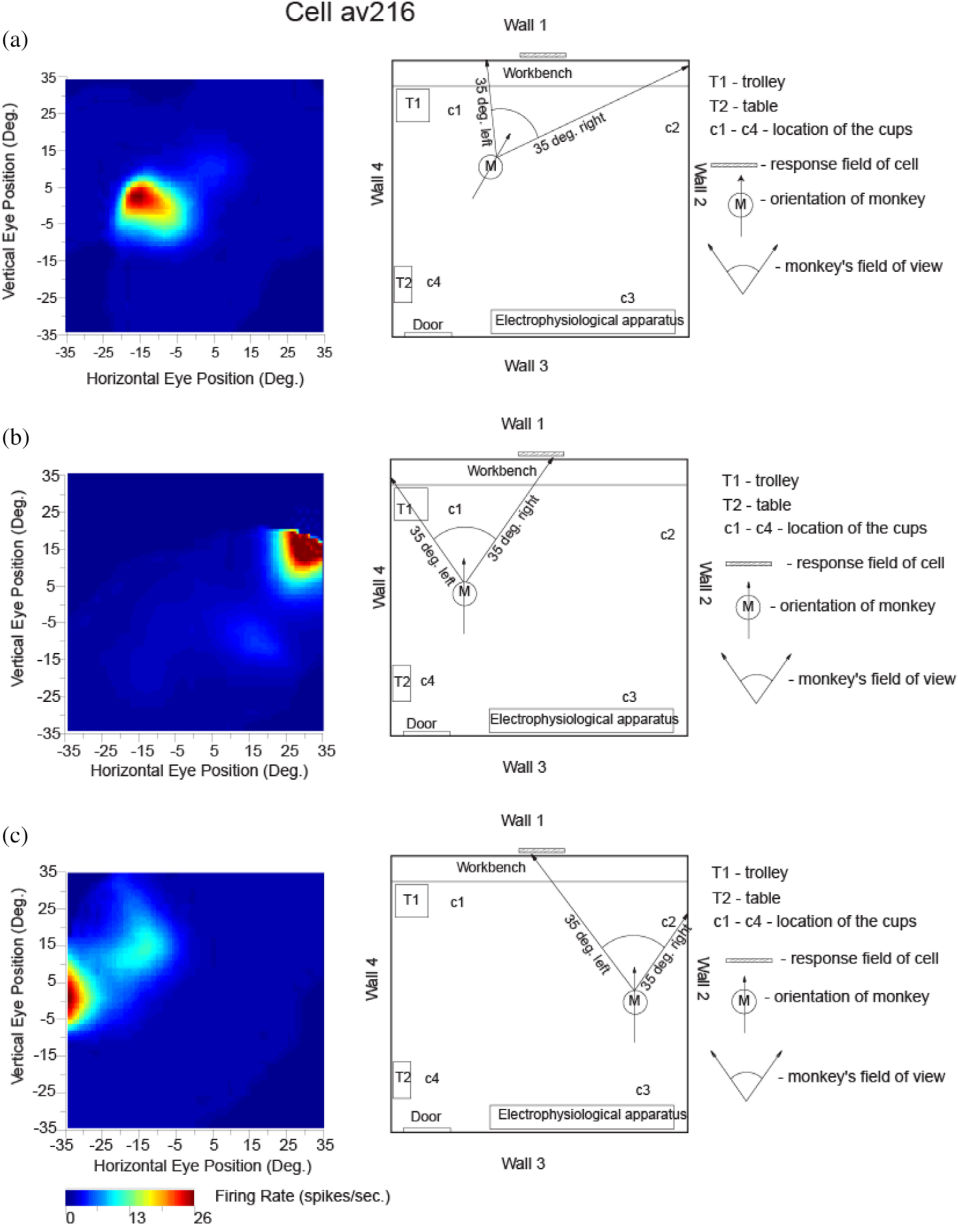
Testing of a hippocampal spatial view neuron (av216) to show that it has allocentric encoding, and that the response does not depend on where the monkey is located. The firing rate is shown as a function of the horizontal and vertical eye position, where positive values indicate right or up. The neuron responded when the monkey looked toward its view field (indicated with a hatched bar) relatively independently of place, eye position, or head direction. ANOVAs and information theory analyses performed on the same data cast in different ways conformed this: For spatial view, the ANOVA was *p* < .001 with 0.217 bits in a 500 ms period for the average Shannon mutual information; for place *p* = .9 with 0.001 bits; for head direction *p* = .5 with 0.0 bits; and for eye position *p* = .8 with 0.006 bits. (Modified from Georges‐François et al. (1999).)

In rodents, place cells respond best during active locomotion (Foster et al., 1989; Terrazas et al., 2005). To test whether place cells might be more apparent in macaques during active locomotion, as active locomotion was thought to be a key issue in rodents (Foster et al., 1989) and may be relevant in primates (Thome et al., 2017), single hippocampal and parahippocampal gyrus neurons were recorded while monkeys very actively walked on all four legs around the test environment with the head and body free to turn (Georges‐François et al., 1999; Robertson et al., 1998; Rolls et al., 1997b; Rolls et al., 1998). Also, to provide a good opportunity for primate hippocampal spatial neurons to reveal how they encoded space, the simple cue‐controlled environment (Rolls & O'Mara, 1995) was changed to a much richer open laboratory environment approximately 5 × 5 m (illustrated in Figures 1 and 2, and with, e.g., windows on walls 1 and 2) within which the macaque had a 2.5 × 2.5 m area in which to walk and forage for food. The place of the monkey and the head direction were tracked continuously while the monkey walked round the environment, and the eye position (which refers to the horizontal and vertical eye directions with respect to the head), were recorded continuously to enable measurement of where the monkey was looking in the environment at all times. The monkey walked round the test area, foraging for food, to enable measurements of neuronal firing for a wide range of places, head directions, and spatial views in a very wide range of different combinations to allow analysis of the relative importance of place, spatial view, and head direction in what was encoded by the neurons (Georges‐François et al., 1999; Robertson et al., 1998; Rolls et al., 1997b; Rolls et al., 1998).

The firing of a hippocampal spatial view cell during active locomotion in this spatial testing environment is illustrated in Figure 1. The neuron fired primarily while the macaque looked at a part of wall three, as emphasized in Figure 1b,c that show a spot on a wall where the monkey was looking when the firing rate was greater than 12 action potentials per s, half of the maximum firing rate. Figure 1b illustrates the finding that the neuron responded while the monkey was looking from different places in the room at the spatial view field on wall 3. The range of different places and head directions over which the hippocampal neuron fired is illustrated in Figure 1c. Analyses showed that this neuron responded to where the monkey was looking in space relatively independently of the place where the monkey was located, and of head direction and eye position. Moreover, the spatial view fields of the neuron were similar when the monkey was actively walking, and also when he was stationary but actively exploring with eye movements different parts of the spatial environment (Georges‐François et al., 1999). Videos to illustrate the firing of spatial view cells are described in the Data Availability Statement at the end of the paper and are provided as Supplementary Material.

The firing of a different hippocampal neuron is shown in Figure 2, to provide evidence, with a different type of analysis, about how the firing is related to spatial view, and not to the place where the macaque is located, or to head direction, or to facing direction, or to eye position. The highest firing of the cell, with the macaque at the place and with the head direction shown in Figure 2a, occurred when the macaque looked 10° left. With the monkey in another place and with a different head direction, the highest firing was when the macaque was looking 30° right, but at the same spatial view (Figure 2b). Figure 2c shows the firing with the macaque at a different place (but the same head direction as in Figure 2b), and the firing was now when the monkey looked approximately 30° left. The spatial view field was at the same place on Wall one as in Figure 2a,b, illustrating the allocentric spatial view encoding provided by spatial view neurons. Examples of video animations to illustrate the firing of macaque hippocampal and parahippocampal spatial view cells are described in the Data Availability Statement and are provided as Supplementary Material.

These experiments show that it is the allocentric spatial view toward which the monkey looks that determines the neuronal responses, and not a particular place where the monkey was located, or head direction, or facing direction, or eye position, and this was confirmed with analyses of variance and with information‐theoretic analyses (Georges‐François et al., 1999; Rolls et al., 1998). It was found that on average the spatial view cells encoded considerably more (mutual Shannon) information about spatial view (0.47 bits) than about eye position (0.017 bits), head direction (0.005 bits), or place in the room (0.033 bits) (Georges‐François et al., 1999). This shows that the encoding by these primate hippocampal neurons may reflect some information about place, and so forth but is primarily about spatial view. The coding is allocentric in that, as illustrated in Figure 2, the neuronal firing occurs when the macaque is looking at a given location in the world, independently of the head direction, facing direction, eye position, and place of the monkey.

The spatial view fields of these hippocampal and parahippocampal spatial view neurons typically occupy a region of space that is approximately as large as 1/16 of all the four walls of the laboratory (Rolls et al., 1998). Each neuron responds to a different view, and the partly overlapping view fields thus provide precise information about the region of space being looked at. Interestingly, some single neurons had more than one spatial view field in this extensive and rich spatial environment (Rolls et al., 1998). Information theoretic measures showed that the information about spatial view increases almost linearly as the number of neurons in the sample increases, thus showing that each of the neurons makes an independent contribution within the population to representing allocentric space (Rolls et al., 1998). Given that Shannon mutual information is a logarithmic measure (Rolls, 2021a; Rolls & Treves, 2011), this evidence indicates that the number of spatial views (or the accuracy of the spatial representation) increases exponentially as the number of neurons in the ensemble increases. This is an important result in terms of how information is encoded by hippocampal and parahippocampal spatial view cells as well as by neurons in other brain areas that encode different types of information (Rolls, 2016a; Rolls, 2021a; Rolls & Treves, 2011). Moreover, this is a firing rate code, with much information present in the number of spikes from a single neuron (Panzeri et al., 1999; Rolls, 2016a; Rolls, 2021a; Rolls & Treves, 2011).

Many hippocampal and parahippocampal spatial view (or “space” or “view”) cells were found in these experiments (Georges‐François et al., 1999; Robertson et al., 1998; Rolls et al., 1997b; Rolls et al., 1998). In the initial sample of 352 neurons recorded under these conditions, the number of spatial view cells was 40, or 11.4% (Rolls et al., 1997b). This was in a single environment (Georges‐François et al., 1999; Robertson et al., 1998; Rolls et al., 1997b; Rolls et al., 1998), and of course, the proportion of neurons would be expected to be higher if testing included several different environments. The spontaneous firing rate of these neurons was low (mean 0.5 spikes/s), and their mean peak firing rate was 17 spikes/s (interquartile range 11–20 spikes/s), consistent with these being hippocampal pyramidal cells, which were in both the CA1 and CA3 regions, with also some spatial view cells in the parahippocampal gyrus.

The finding from rodents that place cells respond better during active locomotion than passive motion (Foster et al., 1989) made it important to investigate primate hippocampal neurons during active locomotion (see also Thome et al., 2017). Having said this, Rolls et al. found that primate hippocampal spatial view cells have similar responses during active locomotion as when the monkey is not locomoting, but is looking around and actively exploring the spatial environment with eye movements. This is shown by the fact that spatial view fields are present when the monkey is stationary as illustrated in Figure 2, or is walking as in Figure 1 (Georges‐François et al., 1999; Robertson et al., 1998; Rolls et al., 1997b); are found when the monkey is tested only when stationary (Rolls et al., 2005; Rolls & O'Mara, 1995; Rolls & Xiang, 2005; Rolls & Xiang, 2006); and as illustrated in the videos described in the Data Availability Statement and provided in the Supplementary Material. Indeed, it is an interesting hypothesis that this active exploration of a spatial environment, by moving the eyes from location to location in a viewed spatial scene in primates, is analogous to the active exploration performed by a rodent when it is locomoting from one place to another place.

### 3.4 Hippocampal spatial view neurons encode allocentric spatial view much more than place or head direction or eye position

To assess whether a neuron in the primate, including human hippocampal system, responds to the place where the individual is rather than spatial view, or head direction, or facing direction, or eye position, extensive testing with contrasts of these different hypotheses is needed (Georges‐François et al., 1999). (Eye position refers to the horizontal and vertical angles of the eye in the orbit.) If the views visible from different places differ, showing that the firing depends on the place where the individual is located is insufficient, because so does the spatial view. To separate spatial view from place cells, neurons must be tested while the individual is in one place with all of the different spatial views visible from there. Further, the same neuron must also be tested when the individual is located in a different place, but with at least many of the same spatial views visible, as has been implemented in Rolls et al.'s recordings in macaques (Georges‐François et al., 1999; Robertson et al., 1998; Rolls et al., 1997b; Rolls et al., 1998). Indeed, although hippocampal neurons in squirrel monkeys were found to respond when the monkeys were in a particular location in a 3D chamber (Ludvig et al., 2004), where the monkeys were looking was not measured, so we cannot contrast spatial view with place coding in this case. Similarly, Ono et al. (1993) found that when a monkey sitting in a cab was moved, some neurons responded when the cab was in specific places in the room. However, they were not able to factor out place from spatial view encoding in the type of factorial design that is necessary. These points will need to be taken into account for future investigations of hippocampal neuronal activity in humans and other primates (cf. Ekstrom, 2015; Ekstrom et al., 2003; Fried et al., 1997; Kreiman et al., 2000; Miller et al., 2013), and recording simultaneously the eye position, head direction, facing direction in the environment, and head position is needed. Only investigations in which the same set of spatial views has been seen from each of the same set of different places can provide evidence about whether the neurons code for spatial view or for place, or perhaps for a combination, and that condition was met in the experiments described above by Rolls et al. Investigations in which different spatial views are present when the individual is in different places do not address the issue about whether the neuronal encoding is about the location being viewed or the place where the individual is located. Of course, if the individual is always in one place, as in some human imaging studies, and neuronal responses are different when different scenes are being viewed, the implication is that the encoding is something about the scene being viewed, and not the place where the individual is.

Having made this point clear, it is nevertheless of interest that for humans there is now some evidence for medial temporal lobe neurons with properties like those of spatial view cells (Ekstrom et al., 2003; Miller et al., 2013), even though direct measures of eye position were not conducted. For example, in the study by Ekstrom et al., cells were found to represent the interaction between the place and the view faced by the patient. It is also of interest that in humans some medial temporal lobe neurons reflect the learning of paired associations between views of places, and people or objects (Ison et al., 2015), and this implies that views of scenes are important for human hippocampal function. Consistent with this, human functional neuroimaging studies do show hippocampal or parahippocampal activation when scenes or parts of scenes are viewed even when the human is fixed in one place for neuroimaging (Brown et al., 2016; Burgess, 2008; Chadwick et al., 2010; Chadwick et al., 2013; Epstein & Kanwisher, 1998; Hassabis et al., 2009; Maguire, 2014; O'Keefe et al., 1998; Stern et al., 1996; Zeidman & Maguire, 2016). Further evidence on the functioning of the human hippocampus is considered in Sections 3.9 and 5.

### 3.5 Idiothetic (self‐motion) update of spatial view cells

**FIGURE 3:**
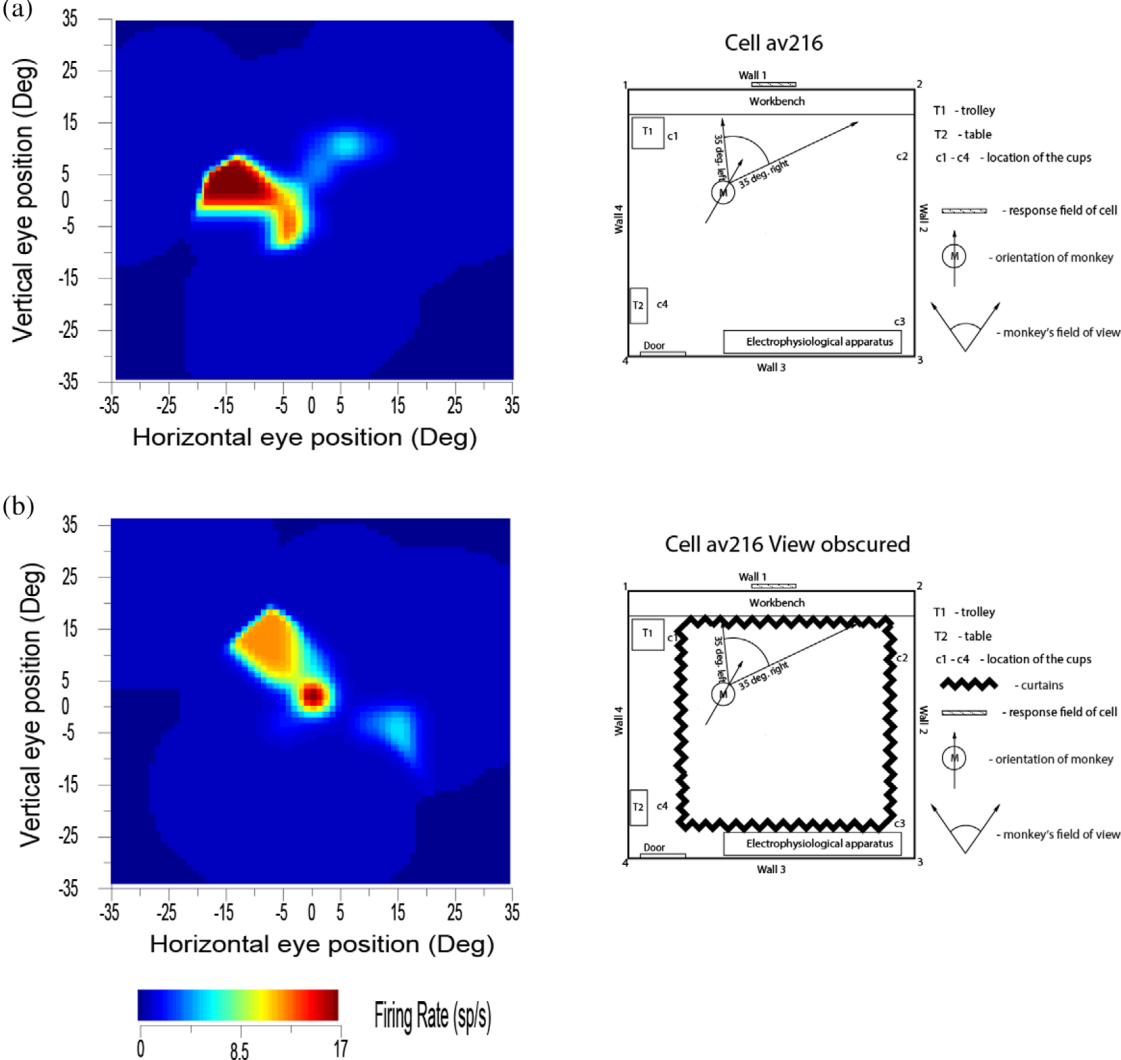
Self‐motion (idiothetic) update of the firing of a hippocampal spatial view cell occurred for a few minutes even when the view details were obscured by floor to ceiling curtains (b). M shows the place of the monkey in the room, with the head direction indicated by the arrow. The self‐motion consisted in the case illustrated of eye movements made by the monkey, but also occurred during locomotion. (Modified from Robertson et al. (1998).)

In rodents, the representation of place by hippocampal place cells can be updated by self‐motion, for example, running in the dark (Jeffery et al., 1997; McNaughton et al., 1991; Quirk et al., 1990). In monkeys, the representation of the location in the scene encoded by spatial view cells can be updated by self‐motion, for example, by the monkey moving the eyes in the dark, or by the monkey turning or walking in the dark. This was shown in experiments on these spatial view cells, in which the view was obscured by black curtains, in which many of the cells could still respond when the macaque moved his eye position to look toward where the view was visible previously (Robertson et al., 1998) (see example in Figure 3). This idiothetic update also occurs when the monkey locomotes in the dark, and then looks to a spatial view location. Some drift of the spatial view field over a few minutes when the curtains were closed was typical, consistent with the hypotheses that self‐motion (idiothetic) updating was occurring, and that the visual view details of the scene normally define the spatial view field of a neuron. It may be remarked that after about the same time in the dark with the curtains closed, the experimenters also lost their updating of place, head direction, and where they were looking in space, and had to find their way out of the environment by feeling for the curtains, and then following them to find a light switch.

These experiments (Robertson et al., 1998; Rolls et al., 1997b) show that primate hippocampal and parahippocampal gyrus spatial view neurons can be updated by self‐motion for short periods by idiothetic information including eye position, head direction, and place movements made by the monkey, and that the drift related to the temporal integration of these signals can be corrected when the scene again becomes visible. These experiments also show that these hippocampal system spatial view cells are different from the much more visual perception‐related responses of inferior temporal visual cortex object and face cells, which stop responding when the object or face is removed from visibility (Rolls, 2003; Rolls & Tovee, 1994).

The neurons had only a small decrease of their response when the room was placed into darkness and/or the view details were obscured with curtains in CA1, the parahippocampal gyrus, and the presubiculum. On the other hand, CA3 neurons had a larger decrease (on average to 23% of their normal response) when the macaque looked toward the normally effective location in the environment but the view was not visible (Robertson et al., 1998). There may be partial recovery of information in the CA3 network using autoassociation (Hasselmo & Wyble, 1997; McNaughton & Morris, 1987; Rolls, 1987; Rolls, 1989a; Rolls, 1989b; Rolls, 2018a; Rolls, 2021a; Rolls & Treves, 1994; Treves & Rolls, 1994), and further recovery in the associative synapses from CA3 to CA1, as has been shown analytically (Schultz & Rolls, 1999) and by simulations (Rolls, 1995). Another contributory factor to the difference might be the direct perforant path input to the CA1 neurons (Rolls, 2016a; Rolls & Treves, 1998).

### 3.6 Population encoding of spatial view by hippocampal neurons

A major issue in computational neuroscience is how information is encoded by populations of neurons, compared to single neurons (Rolls, 2021a; Rolls & Treves, 2011). For example, how does the number of stimuli, for example, spatial view locations, that can be encoded increase with the number of recorded neurons? To investigate this, we applied Shannon mutual information theoretic techniques useful for analyzing neuronal responses (described in detail elsewhere (Rolls, 2021a; Rolls & Treves, 2011) with code made available (Rolls, 2021a)) to the responses of macaque hippocampal spatial view neurons (Rolls et al., 1998). First, it was found that different hippocampal spatial view neurons tended to have different spatial view fields. It was then found that the information from an ensemble of these neurons about the 16 viewed locations increases approximately linearly with the number of cells in the population, which in this experiment was 20 neurons (Rolls et al., 1998). This indicates that the neurons convey independent information about spatial view (up to this number of neurons), and therefore, as information is a log measure, that the number of locations that can be encoded increases exponentially with the number of neurons in the population (Rolls, 2021a; Rolls et al., 1998). In information theory terms, this means that the “signal correlations,” that is, the correlations between the response profiles of each neuron to the set of stimuli, are low (Rolls, 2021a). An important further result was that when the decoding procedure for how the neurons encode location that was used to measure the information was made very biologically plausible, decoded by just a dot product of the firing of the neuronal population, then the information was almost the same. This type of decoding could be performed by the simplest model of a neuron that linearly sums the activity of each of its inputs, which is the simplest operation a neuron could perform (Rolls, 2021a). This makes the whole analysis biologically plausible. Decoding procedures that use non‐biologically plausible algorithms (Diamanti et al., 2021; Panzeri et al., 2022) may overestimate the information that is actually available for use by neurons in the brain.

In this investigation, the hippocampal neurons were not recorded simultaneously, and it is just possible that if the firing of the different neurons was cross‐correlated in time for some but not other stimuli (locations), then extra information might be available from these so‐called “noise correlations” (Panzeri et al., 2022; Rolls, 2021a). However, although that remains to be tested for primate hippocampal neurons, this is rather unlikely, for when macaque inferior temporal cortex neurons responding to faces or objects are simultaneously recorded and the effects of any possible noise correlations are tested, any effects found are small, and very much less than the large amount of information available from the number of spikes, that is, from the measured firing rates (Aggelopoulos et al., 2005; Franco et al., 2004; Franco et al., 2007; Rolls, 2021a; Rolls et al., 2004; Rolls, Franco, et al., 2003; Rolls, Franco, et al., 2006; Rolls & Treves, 2011). Indeed, stimulus‐dependent (i.e., “noise”) correlations between neurons may typically reduce the amount of information that is available from a population of neurons depending on how it relates to the signal correlations (Cohen & Kohn, 2011; Kanashiro et al., 2017; Panzeri et al., 2022; Ruff & Cohen, 2016), rather than perhaps solve the binding problem as had been previously suggested (Engel et al., 1992; Kreiter & Singer, 1996; Singer, 1999).

A number of approaches have analyzed population encoding in especially situations where the space being encoded is low‐dimensional, in, for example, the motor system, and the question has been raised of whether non‐linear decoding by neurons, which might not be very biologically plausible, might be used (Ebitz & Hayden, 2021; Keemink & Machens, 2019; Kriegeskorte & Wei, 2021; Saxena & Cunningham, 2019). But low‐dimensional spaces are not typical for the encoding of information in the cortex, where we may, for example, be able to recognize 10,000 different objects, thousands of spatial locations across many scenes, in the order of 10,000 episodic memories in the hippocampus, and so forth (Rolls, 2018a; Rolls, 2021a; Rolls & Treves, 2011). In this situation, a very large number of synapses on each neuron is required, in the order of 10,000 as is found, and the 10,000 dimensionality of this space is sufficient for these numbers of objects, locations, or episodic memories to be stored and later retrieved correctly, from autoassociation attractor memories and from pattern association memories (Rolls, 2021a; Rolls & Treves, 1990; Treves & Rolls, 1991). For this much more usual type of neuronal encoding of high‐dimensional spaces, the decoding can be linear, which is biologically plausible, and the computationally useful nonlinearity is introduced by the operation of competitive networks (Rolls, 2016a; Rolls, 2021a), such as those believed to be present in the dentate gyrus and CA1 (Rolls, 2016a; Rolls, 2018a; Rolls, 2021a; Rolls & Mills, 2019; Rolls, Stringer, & Elliot, 2006), as well as in the neocortex (Rolls, 2021c).

### 3.7 Spatial representations in primates in a virtual environment

Virtual environments can be used to investigate spatial representations, and have advantages that the recordings can be made more easily than during navigation through real environments, and disadvantages that there are no vestibular and proprioceptive inputs related to motion, and at least in macaques and humans the body movements are likely to be very different (Minderer et al., 2016).

Macaque hippocampal neurons that respond to the place where the individual is located have been described in a virtual navigation task (Furuya et al., 2014; Hori et al., 2005), and some neurons also appeared to be related to view, but eye position recording was not available.

Eye position was recorded from macaques in a virtual navigation task in a star maze with five landmarks and a reward hidden between two of the landmarks (Wirth et al., 2017). It was reported that 28% (53/189) of hippocampal cells fired when the animals looked at one or sometimes more than one of the landmarks, and that 83% of these cells had their responses modulated by place. Then, 17% of the hippocampal neurons responded when the macaque was in one or more places in the virtual environment without significant modulation by where the animal was looking. Some neurons responded to combinations of view, place, and task context (Wirth et al., 2017).

The identification of neuronal responses that depended on where the animal was looking in a virtual navigation task was very interesting (Wirth et al., 2017). So was the discovery that some of the neurons responded when the monkeys moved their eyes to a new spatial location just before the view appeared on the screen in the VR task (Wirth et al., 2017). This provided clear evidence for idiothetic update of view‐related responses, with the idiothetic movement in this case the eye movement.

The study was also interestingly extended by showing that when the star maze and goal remained the same, but five new landmark cues were substituted, the hippocampal neurons quickly learned to respond to the new visual cues used for the landmarks (Baraduc et al., 2019). An interpretation is that the topological chart of the environment previously learned for the maze which links the landmarks, goal, and places in the environment (Battaglia & Treves, 1998) could remain relatively unaltered, and new room landmarks cues could be rapidly associated onto the existing chart of the star maze.

Some neurons with spatial view properties have activity that is modulated by the place where the individual is located (Rolls & O'Mara, 1995; Wirth et al., 2017). For example, the finding that many of the primate hippocampal neurons that responded to the sight of landmarks in a star maze did so mainly from certain places might have been because the navigational task constrained the individual to stay within the star maze, so that looking at some landmarks from some places, and with a particular view of the star maze in the foreground, might have not provided the opportunity for the landmarks to be seen in the same way from all places (Wirth et al., 2017), in contrast to the testing in an open field used by Rolls et al. (Georges‐François et al., 1999; Robertson et al., 1998; Rolls et al., 1997b; Rolls et al., 1998). Effectively, it could be that in a maze, in which only certain combinations of spatial view, place, body turn, and so forth can occur, neurons can learn to respond only to whatever conjunctions of sensory input actually occur. Instead, in the real world and in an open field, neurons may be much better able to learn spatial view representations that are invariant with respect to place because the spatial view can be seen from many different places. In rodents, something like this does happen in a hairpin maze in which the places, head directions, and views are constrained by the maze (Derdikman et al., 2009). This prevents the formation of the usual 2D place fields of hippocampal neurons found in open field environments, and instead results in place cells firing at only some parts of the hairpin track being followed, and probably representing the few combinations of place, head direction and view afforded by the hairpin maze (Derdikman et al., 2009). Another and interesting possibility is that some of these neurons only responded to a landmark when it was at a particular bearing, which is how an “allocentric bearing to a landmark” cell would respond (Rolls, 2020). Another possibility is that testing in a maze emphasizes looking at certain landmarks from certain places, as this is important for the navigation task (Wirth et al., 2017), so the type of combination representation just described of view, place, and head direction may have been formed because that was the sensory input provided in the maze. Indeed, it is further postulated that in the same environment, if a primate (including a human) is navigating to a place, as contrasted to navigating to a series of landmarks, the task demands and the inputs reaching the individual may have a modulatory influence on the extent to which spatial view versus place encoding is evident in the hippocampal system. This would be interesting to explore in future research. Outside highly learned and fixed navigational tasks, spatial view cells that are relatively invariant with respect to place are likely to be more important, for then the viewed location toward which one is navigating can be recognized independently of the current place of the individual (Rolls, 2021b).

In another series of investigations during virtual navigation in macaques, it has been found that the spatial properties of hippocampal neurons can be influenced by whether a task is being performed, though in this case it was not clearly possible to separate place from viewed location encoding (Gulli et al., 2020). For comparison and in contrast, in real‐world tasks in which macaques had to learn associations between view locations and objects or rewards, it was found that the spatial encoding was stable, in that the viewed location in the laboratory to which a spatial view cell was responding first had to be identified, and then with that location included in the experiment, associations of objects or rewards with that spatial view location could be identified (Rolls et al., 2005; Rolls & Xiang, 2005).

In another virtual reality investigation in macaques, of 88 hippocampal neurons with selective spatial responses, 32 responded to view, 12 to place, and 44 to both (Tan et al., 2021; Yen & Tan, 2023).

### 3.8 Encoding of allocentric spatial view compared to facing direction

Useful confirmation has also recently been obtained that relatively many macaque hippocampal neurons respond to the location “out there” in space toward which the animal is facing (22% of neurons), compared to only 5% of hippocampal neurons that encode the place where the macaque is located (Mao et al., 2021). Some neurons were classified as spatial view cells and others as “facing location” cells, but the environment being viewed was simple (a cylindrical arena with a drain on the floor and two touchscreens with food on the walls), and more spatial view cells are likely to be found in a rich spatial environment such as the open lab that we used (Georges‐François et al., 1999; Robertson et al., 1998; Rolls et al., 1997b; Rolls et al., 1998). Indeed, the reason that we moved to a rich open lab visual environment was that we expected to find, and did find, more spatial view cells than in a relatively simple spatial environment with only four cues in the testing arena (Rolls & O'Mara, 1995). Spatial view cells in our testing environments were found to respond to where the macaque was looking in space, and not to the location toward which the individual was facing, by testing these specific hypotheses (Georges‐François et al., 1999; Rolls et al., 1997b; Rolls et al., 1998; Rolls & O'Mara, 1995). A clear example showing that spatial view cells of the type that we have described code for where the monkey is looking in allocentric space and not where he is facing is shown in Figure 2. Further evidence is that in the dark, spatial view cells respond to a remembered spatial view location only when that location is being looked at, with facing location held constant (Robertson et al., 1998) (Figure 3).

In terms of brain computations, it is computationally useful for spatial view cells to respond to viewed allocentric locations in a natural scene that has many useful and clear landmarks, even if an individual is not facing those locations but is looking at them, because it is where objects or landmarks are in the real world, not where one is facing, that is important for memory of where objects are in the world and navigation to a location in the world (Rolls, 2021a). To make this point very clear, a representation that depends on facing direction (Mao et al., 2021) is not very useful because it is not invariant with respect to place. That is, to find an object or reward in a scene, the individual has to go to the place where the memory was formed, and then when facing in the correct direction, facing location neurons would fire and the object or reward associated with that could be retrieved from memory. In contrast, spatial view cells are allocentric, and invariant with respect to eye position, head direction, and place where the individual is located, so that wherever the individual is placed, a spatial view cell will respond, and potentially enable the recall of the object or reward at that viewed location (Georges‐François et al., 1999; Rolls & O'Mara, 1995). That true allocentric representation provided by hippocampal and parahippocampal spatial view cells is thus much more useful than a representation that is based on “facing location.” Of course, when primates navigate they may often be facing in the direction in which they are navigating. But that does not mean that allocentric spatial view is not being encoded, and experiments of the type illustrated in Figure 2 show clearly that allocentric spatial view is being encoded by spatial view neurons (Georges‐François et al., 1999; Rolls et al., 1997b; Rolls et al., 1998; Rolls & O'Mara, 1995).

### 3.9 Spatial view cells in humans

For humans, there is evidence for medial temporal lobe and hippocampal neurons with properties like those of spatial view cells, for example, with responses to locations being viewed (from recordings in patients during neurosurgery) (Ekstrom et al., 2003; Miller et al., 2013). In the study by Ekstrom et al. (2003), some medial temporal lobe neurons were found to represent views of landmarks. In another study of human medial temporal lobe neurons, it was found that in a Treasure Hunt game, some neurons respond to the sight of remote locations rather than the subject's own place (Tsitsiklis et al., 2020). Just like macaque spatial view cells, these neurons in humans respond when the spatial location is seen with different bearings (showing that they are not “allocentric‐bearing‐to‐a‐landmark” neurons, but spatial view neurons). The locations in the human Treasure Hunt game were in at least some cases within the spatial environment that could be viewed. In the macaque testing, hippocampal spatial view neurons could respond when the macaque was distant from an effective part of the 3D environment (e.g., the location in the scene where a trolley was located), but also when the macaque was close to the effective part of the environment (e.g., at the place where the trolley was located, as illustrated by Rolls (1996a, 2021a)). This is thus somewhat comparable to the way in which the human visual “spatial target” neurons responded (Tsitsiklis et al., 2020). The results in humans (Tsitsiklis et al., 2020) thus appear to confirm the presence of spatial view cells in humans that were discovered in macaques (Feigenbaum & Rolls, 1991; Rolls et al., 1989; Rolls et al., 1997b; Rolls & O'Mara, 1995). In addition, some neurons have been recorded in humans that respond during navigation toward the location of a particular goal in a virtual environment (Qasim et al., 2019; Qasim et al., 2021). Further, in humans, some medial temporal lobe neurons reflect the learning of paired associations between views of places, and people or objects (Ison et al., 2015) (just as in macaques (Rolls et al., 2005)), and this implies that neurons coding for views of scenes are important for human hippocampal function.

Consistent with this, human functional neuroimaging studies do show hippocampal or parahippocampal activation when scenes or parts of scenes are viewed even when the human is fixed in one place for neuroimaging (Brown et al., 2010; Brown et al., 2016; Burgess, 2008; Chadwick et al., 2010; Chadwick et al., 2013; Epstein & Kanwisher, 1998; Hassabis et al., 2009; Maguire, 2014; O'Keefe et al., 1998; Zeidman & Maguire, 2016). That is further evidence that representations of space “out there” are key to hippocampal function in primates including humans. Further, using an adaptation paradigm while participants viewed snapshots of a virtual room which differed in place, spatial view, and heading, it was found that the pattern of hippocampal activity reflected both view‐based and place‐based distances, the pattern of parahippocampal activity preferentially discriminated between views, and the pattern of retrosplenial activity combined place and view information (Sulpizio et al., 2014). Using a somewhat similar approach, evidence for encoding of heading direction in the human presubiculum was found (Vass & Epstein, 2013), consistent with the head direction cells found in the macaque presubiculum (Robertson et al., 1999).

In the human parahippocampal gyrus, neurons have also been described in virtual navigation that represent egocentric directions toward “anchor points” (Kunz et al., 2021), which are similar to the landmarks described in Sections 5, 6 (Rolls, 2020). Some of these neurons also encoded the distance to the landmarks. Neurons of this type may be part of the interface to actions performed in space in which the human parietal cortex which has connectivity with the parahippocampal gyrus (Rolls, Deco, et al., 2022c; Rolls, Deco, et al., 2022d; Rolls, Wirth, et al., 2022) is important (Sections 5.3 and 8). In humans, an fMRI investigation showed that the distance to home may be represented in the hippocampus and retrosplenial cortex (Chrastil et al., 2015). Some other parahippocampal gyrus neurons encoded allocentric direction (such as “West”) (Kunz et al., 2021), and might be similar to the head direction cells described in Section 3.13 (Robertson et al., 1999).

### 3.10 Comparison of primate hippocampal spatial view cells with other types of neuronal response

To further elucidate the properties of primate hippocampal spatial view cells, they are now compared to object cells in the macaque inferior temporal visual cortex, and then to “concept” cells in humans, and then to head direction cells.

Hippocampal spatial view cells are quite different to inferior temporal visual cortex (IT) cells that respond to faces or objects (Aparicio et al., 2016; Arcaro & Livingstone, 2021; Booth & Rolls, 1998; Freedman, 2015; Freiwald, 2020; Freiwald et al., 2009; Hasselmo et al., 1989; Perrett et al., 1979; Perrett et al., 1982; Rolls, 2000; Rolls, 2021a; Rolls, 2021f; Rust & DiCarlo, 2010; Tsao, 2014) wherever they are moved to in a spatial environment (Aggelopoulos et al., 2005; Rolls, 2012; Rolls, 2016a; Rolls, Aggelopoulos, & Zheng, 2003). On the other hand, it is normally the combination of a set of features in a fixed position relative to each other in the world that activates spatial view neurons (Feigenbaum & Rolls, 1991). A helpful distinction is that objects can be moved to different places in the environment, and visual temporal cortex object‐selective neurons respond to an object independently of its location in a scene (Aggelopoulos et al., 2005; Rolls, Aggelopoulos, & Zheng, 2003). In contrast, parts of a spatial scene are fixed with respect to other parts of the scene, and cannot be moved independently with respect to the other parts. Thus, although hippocampal spatial view cells can respond to stimuli that are a fixed part of a spatial scene (e.g., table T2 in Figure 1 of Robertson et al. (1998)), the point is that this is a fixed part of a continuous spatial scene. Spatial scene representations may be learned by associating together features in a scene that have a fixed spatial relationship to each other (Rolls & Stringer, 2005; Stringer et al., 2005), and this is quite different from invariant visual object learning in which the features of a single object are associated together, because of regular association of the parts of a single object that are independent of the background scene and other objects in the scene that are not constant (Rolls, 2012; Rolls, 2016a; Rolls, 2021f; Stringer et al., 2007; Stringer & Rolls, 2008). The inputs to the hippocampal formation that help it to form spatial view representations may come from areas such as the occipital place area (Julian et al., 2016), and from scene processing areas in the macaque temporal cortex (Kornblith et al., 2013) and human PSA (Rolls, Deco, et al., 2022b).

Another difference from IT neurons is that many hippocampal spatial view neurons, because they represent parts of space, can respond even when the scene is not visible but that part of space is looked at (Rolls et al., 1997a); and can be updated idiothetically, that is by self‐motion, in that spatial view neurons respond when a macaque moves the eyes to a location in space even when no scene is visible, and in darkness (Rolls et al., 1997a) (see Section 3.5).

Another difference is that IT neurons respond well to visual stimuli in an object‐reward association task (Rolls, Aggelopoulos, & Zheng, 2003; Rolls et al., 1977), but hippocampal neurons have weak responses to objects in this type of non‐hippocampal‐dependent task, compared to the stronger object‐related responses that can occur in an object‐place, hippocampus‐dependent, task (Rolls & Xiang, 2005).

In primates, hippocampal neurons have been described that respond in an invariant way to the sight of individual faces (Quiroga et al., 2005; Sliwa et al., 2016). The neurons found in humans described as “concept cells,” an example of which is a neuron that responded to Jennifer Aniston, may respond not only to Jennifer Aniston, but also to other actors in the same movie, and the places with which they are associated (De Falco et al., 2016; Quiroga, 2012; Quiroga et al., 2005; Rey et al., 2015). *Object‐place cells in macaques have some similar “concept” properties, in that they can be activated either by the object, or by the place, in object‐place memory tasks* (Rolls et al., 2005; Rolls & Xiang, 2006). Similar properties have been described for human hippocampal neurons, in a task in which a human was associated with a place (Ison et al., 2015).

It can also be emphasized that spatial view hippocampal and parahippocampal cells are quite distinct from head direction cells, found in the primate presubiculum and parahippocampal gyrus (Robertson et al., 1999). For instance, if the head direction remains constant when the macaque is moved to different places in the environment where the spatial view differs, spatial view cells provide different responses. On the other hand, head direction cells have activity that remains constant for a particular head direction, even though the spatial view differs completely (Robertson et al., 1999).

### 3.11 Grid cells in rodents and spatial view grid cells in the primate entorhinal cortex

In the rodent entorhinal cortex, grid cells that represent places by hexagonal place grids and are involved in idiothetic update of place have been described (Gerlei et al., 2021; Kropff & Treves, 2008; Moser et al., 2015). In macaques, a grid‐cell like representation in the entorhinal cortex has been found, but the neurons have grid‐like firing as the monkey moves the eyes across a spatial scene (Garcia & Buffalo, 2020; Killian et al., 2012; Meister & Buffalo, 2018; Rueckemann & Buffalo, 2017). Similar competitive learning processes to those suggested for rodents (Rolls, Stringer, & Elliot, 2006) may transform these primate entorhinal cortex “spatial view grid cells” into primate hippocampal spatial view cells (Rolls, 2021a), and may contribute to the idiothetic (eye movement‐related) update of spatial view cells (Robertson et al., 1998). The existence of spatial view grid cells in the entorhinal cortex of primates is predicted from the presence of spatial view cells in the primate CA3 and CA1 regions (Kesner & Rolls, 2015; Rolls, 2013; Rueckemann & Buffalo, 2017). Moreover, some of these “spatial view grid cells” have their responses aligned to the visual image (Meister & Buffalo, 2018), as predicted (Kesner & Rolls, 2015).

In the human entorhinal and cingulate cortex neurons with grid‐like response properties are found (Jacobs et al., 2013; Nadasdy et al., 2017), and there is neuroimaging evidence that is consistent with this (Julian et al., 2018; Nau et al., 2018). This is further evidence for the concept that representations of locations being viewed in space “out there” are a key property of spatial representations in the hippocampal system of primates including humans.

### 3.12 Neurons useful for idiothetic update of spatial view neurons: Primate hippocampal whole body motion neurons

To perform idiothetic update of a spatial representation (such as that provided by spatial view or place cells), a self‐motion signal is needed to update the spatial representation. The idiothetic signal might usefully be a velocity of movement signal. This velocity signal might have its origin in vestibular signals about motion, in optic flow, and/or in corollary motor discharge (Bremmer, Duhamel, et al., 2002; Bremmer, Klam, et al., 2002). Neurons that do respond to self‐motion signals have been discovered in the primate hippocampus, in an investigation in which the monkey was moved while sitting on a robot with defined axial rotations and linear translations, and in a test situation in which optic flow visual motion cues could also be produced by rotating the whole environment round the monkey (O'Mara et al., 1994). The neurons respond to the velocity of whole body motion (O'Mara et al., 1994), which is idiothetic information. For instance, some neurons have larger responses for clockwise than for anti‐clockwise whole body rotation. Occlusion of the visual field showed that some of these neurons depend on visual input. For other neurons, there was no requirement for visual input, and these neurons probably responded to vestibular input. Other neurons responded to a combination of whole‐body motion and view or place. Of the 45 neurons with responses related to whole body motion (9.8% of the population of hippocampal neurons recorded), 13 responded to axial rotation only, 9 to linear translation only, and 20 neurons to axial rotation or to linear translation. The sign of the motion was important for some of the neurons, with different responses for clockwise versus anticlockwise rotation, or for forward versus backward linear translation, which are different velocities. Some neurons responded to a combination of whole body motion and either a local view (*n* = 2) or a place toward which the macaque was moving (*n* = 1).

Whole‐body motion neurons are likely to be a useful component of a memory system for memorizing spatial trajectories through environments for path integration that is useful in short‐range spatial navigation (O'Mara et al., 1994). They may provide self‐motion information useful to provide the idiothetic update of spatial view cells. Consistent with this discovery (O'Mara et al., 1994), neurons have more recently been found in the rat entorhinal cortex that have a linear response with linear running speed, and have been termed “speed cells” (Hinman et al., 2016; Kropff et al., 2015).

### 3.13 Neurons useful for idiothetic update of spatial view neurons: Primate head direction cells

A second principal type of neuron in primates that provides idiothetic information useful for the update during self‐motion of spatial view neurons (Robertson et al., 1998), as well as for navigation, is head direction cells, well known in rodents (Cullen & Taube, 2017; Taube et al., 1990), which we discovered in the primate presubiculum (Robertson et al., 1999) (and they are probably elsewhere). Head direction neurons are likely to be important in updating spatial view cell representations of allocentric space and for navigation when the view details are obscured or in the dark (Rolls, 2020; Rolls, 2021b).

Head direction neurons continue to encode head direction even when the monkey is moved from a familiar room to a relatively unfamiliar corridor, and maintain their directionality for a few minutes in the dark, after which they drift (Robertson et al., 1999). This is important, for these cells can only maintain head directionality for a relatively short period without visual cues to lock them back into the correct directionality. Their inputs are derived from velocity signals produced in the vestibular nuclei in the brainstem and reach the parietal vestibular cortical areas (Cullen, 2019; Grusser et al., 1990; Ventre‐Dominey, 2014). The direction signal thus reflects a great deal of integration over time, and this is imprecise and noisy resulting in drift. This means that only short‐term idiothetic navigation (i.e., without visual cues) is possible. Vestibular signals influence neurons in a number of parietal cortex areas including VIP, with neurons that respond to head position (i.e., head direction) or head acceleration, in addition to the many neurons with head velocity tuning (Klam & Graf, 2003). Neurons that respond to vestibular inputs produced by head rotation or translation are also found in area 7a (Avila et al., 2019). The parietoinsular vestibular cortex may be especially important in the sense of direction (Chen et al., 2016).

## 4 PRIMATE HIPPOCAMPAL ALLOCENTRIC SPATIAL VIEW NEURONS, AND OBJECT‐LOCATION AND REWARD‐LOCATION EPISODIC MEMORY

Primates have a highly developed ventral stream cortical visual system that utilizes information from the fovea for object recognition, and a highly developed eye movement control system to bring the fovea to objects, using mechanisms described elsewhere (Rolls, 2012; Rolls, 2016a; Rolls, 2021a; Rolls, 2021f; Rolls, Aggelopoulos, & Zheng, 2003; Rolls, Deco, et al., 2022b; Rolls, Deco, et al., 2022c; Rolls & Webb, 2014). These developments enable primates to explore and remember information about what is present at locations seen “out there” in the spatial environment without having to visit those places. Spatial view cells would therefore be useful as part of a memory system by providing a representation of space that does not depend on where the primate is, and that could be associated with items such as objects or rewards in those viewed spatial locations. This could enable a monkey to remember where it had seen ripe fruit, or a human to remember where in a spatial scene they had seen a person. Primate hippocampal system spatial view neurons may therefore be important in forming memories of what has been seen and where it has been seen even on a single occasion, a key component of an episodic memory. Episodic memories of this type would be useful for spatial navigation or action in space, for which according to Rolls' hypothesis the hippocampus would implement the memory but not necessarily the spatial computation component (Kesner & Rolls, 2015; Rolls, 2020; Rolls, 2021b; Rolls, 2022), with evidence for this provided in Section 11, and in Section 5.4 of Rolls and Wirth (2018).

Evidence is now described that these hippocampal allocentric spatial view neurons have activity that is involved in episodic memory‐related spatial functions. It is noted that neurons with egocentric or facing direction sensitivity (Mao et al., 2021) would be much less useful in an episodic memory system, for such neurons would only be useful for recall when the egocentric or facing measures occurred again. That is much less useful and general when memorizing where an object or reward is in allocentric world‐based coordinates, for then the object or reward associations of that location in the world can be recalled independently of the facing direction of the individual or the place, head direction, eye position, and retinal position of the individual.

### 4.1 Object‐spatial view location neurons in the primate hippocampus

A key issue is whether the primate including human hippocampus is for memory, or for navigation. There is emphasis on navigation for place cell function in rodents (Burgess et al., 2000; Burgess & O'Keefe, 1996; Hartley et al., 2014; O'Keefe, 1979; O'Keefe, 1991). However, the hippocampus is implicated in episodic memory in which the location, or temporal position in a sequence of a single episodic memory, is associated with, for example, the associated objects or rewards (Dere et al., 2008; Eichenbaum et al., 2012; Hasselmo, 2009; Kesner & Rolls, 2015; Rolls, 1990; Rolls & Mills, 2019; Treves & Rolls, 1994; Zeidman & Maguire, 2016). If the hippocampus helps to implement episodic memory, then object information would need to reach the hippocampus, where it might be combined with spatial view information to form for example, an episodic memory of a person or object seen in a viewed location.

To investigate the fundamental issue of whether object information, as well as spatial view information, is provided in the primate hippocampus, single hippocampal neurons were recorded during an object‐place memory task in which the monkeys had to learn associations between objects and where they were shown in an open laboratory (Rolls et al., 2005). Some neurons (10%) responded to an object independently of its location; other neurons (13%) responded to spatial view independently of the object shown; and some neurons (12%) fired to a combination of a particular object and the particular location where it was shown in the laboratory. Thus, in the primate hippocampus, there are separate as well as combined representations of objects and of their locations in space. These properties are needed in an episodic memory system, for associations between objects and where they are seen are prototypical for episodic memory. These discoveries provide evidence that a key requirement for a human episodic memory system, both separate and combined neuronal representations of objects and their locations “out there,” are present in the primate hippocampus (Rolls et al., 2005). These neurons might also be termed object‐spatial view neurons, to emphasize the difference from what is found in rodents. Neurons that correspond have now been described in rodents, but they, as expected, encode item‐place, not item‐spatial view, combinations (Komorowski et al., 2009). In the rodent investigation, the items were odors.

Consistent with these discoveries, the hippocampus receives projections from the temporal cortical areas specialized for objects or faces (Huang et al., 2021; Perrett et al., 1982; Rolls, 2000; Rolls, 2021a; Rolls, 2021f;Rolls, Deco, et al., 2022b; Rolls, Deco, et al., 2022d), and neurons responsive for particular individuals were found both in the human medial temporal lobe (Kreiman et al., 2000; Quiroga, 2012) and the monkey hippocampus (Sliwa et al., 2016).

### 4.2 One‐trial, object‐spatial view, recall‐related neurons in the primate hippocampus

**FIGURE 4:**
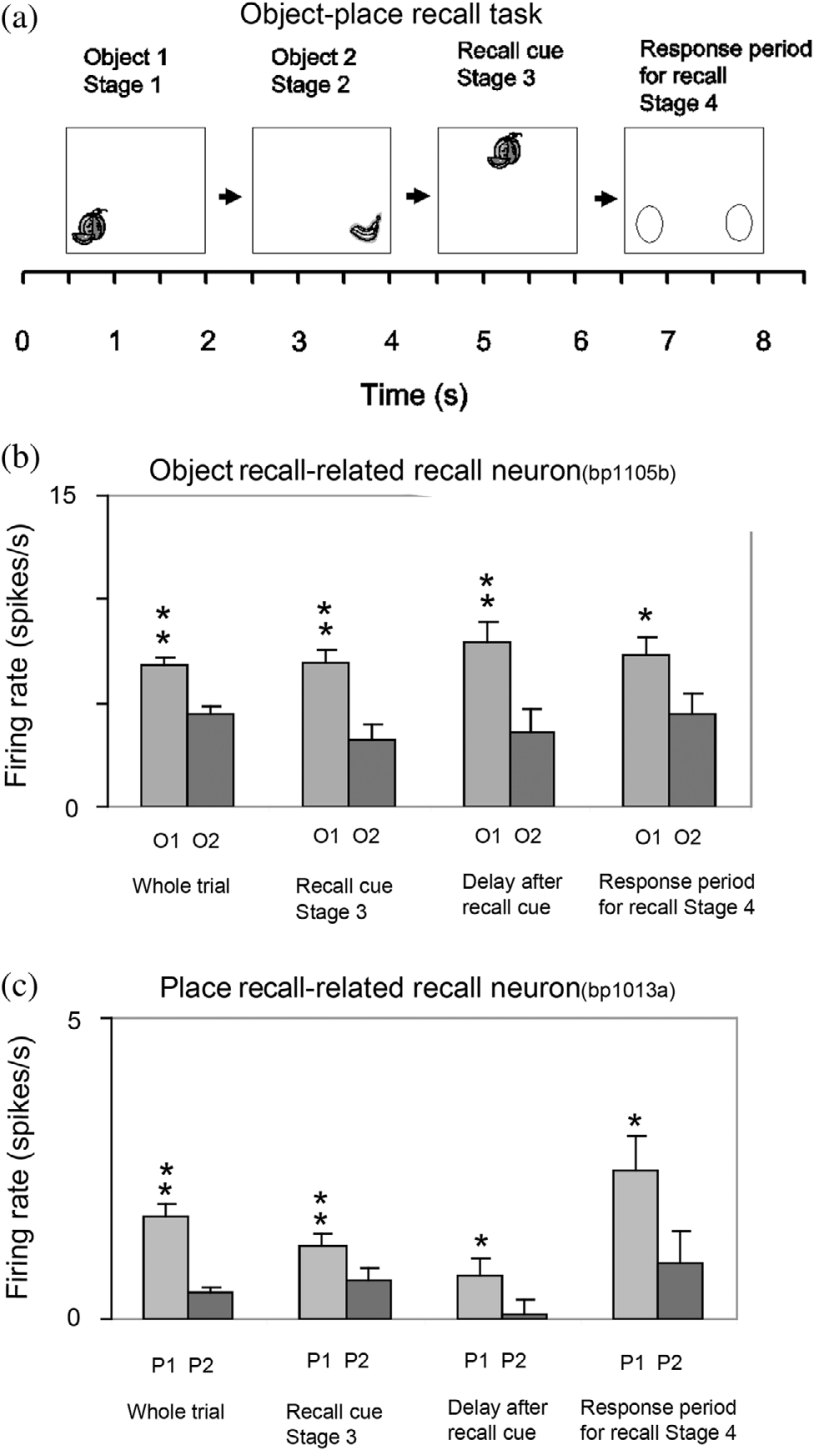
Firing of hippocampal neurons in a one trial object‐viewed location recall task. (a) In stage 1, object 1 was shown in a location on the screen being viewed, and in stage 2 object 2 was shown. In stage 3, one of the objects was shown at the top center of the screen, and the monkey had to touch the location on the screen where that object had been shown in order to obtain a juice reward. (b) A neuron that was selective for object 1 (O1) responded even in stage 4 when the object was not visible but the object and its location had to be recalled. (c) A neuron that was selective for location 1 (P1) responded even in stage 4 when the object was not visible but the object and its location on the screen being viewed had to be recalled. The average firing rate in spikes/s across trials ± *SEM* is shown. **p < .01; *p < .05. (Modified from Rolls and Xiang (2006).)

A feature of the theory of the hippocampus in episodic memory is that object and location memories should be capable of being formed in one trial, in order to be relevant to the timescale of episodic memory, and that the whole memory can be recalled from any part (Kesner & Rolls, 2015; Rolls, 1989b; Rolls, 1996b; Rolls, 2016a; Rolls, 2018a; Rolls, 2021a; Rolls & Kesner, 2006; Treves & Rolls, 1994). This was tested in macaques in a one‐trial memory task in which a viewed object had to be associated with its viewed spatial location. The task involved the storage of object‐location information, and then the recall of the object when the location was presented as a recall cue, and the recall of the location when the object was presented as a recall cue (Figure 4a). The design is similar to that of a one‐trial odor‐place recall memory task that is hippocampal‐dependent in rats (Day et al., 2003), and is quite different from a long‐term visual–visual associative memory task which is implemented in the perirhinal and related cortex (Fujimichi et al., 2010; Hirabayashi et al., 2013; Naya et al., 2001). Images of novel objects were used every day, and within a day, the same objects were used, so that the one‐trial recall task was difficult. Recordings were made from 347 hippocampal neurons during the performance of the object‐location memory storage and recall task (Rolls & Xiang, 2006). Some neurons performed object recall, when the recall cue was a viewed location (Figure 4b). Some neurons performed location recall, when the recall cue was an object (Figure 4c). The recall‐related firing is evident in stage 4, when the object or location was being recalled with no stimulus present on the screen. Details of the results are provided elsewhere (Rolls & Xiang, 2006). The findings provide evidence that the macaque hippocampus can provide for one‐trial object‐view association learning of the type that is prototypical for episodic memory (Rolls & Xiang, 2006). Rapid changes in neuronal response properties as a result of learning associations between items such as individuals and places have been confirmed in humans (Ison et al., 2015).

In humans, in an object‐place recall task in virtual reality, some neurons during recall also reflect the recall of the place when the object recall cue is provided (Miller et al., 2013). Further, it has been shown that time as well as space is encoded in the primate hippocampus, and that some hippocampal neurons recalled the time at which a specific object was seen (Naya & Suzuki, 2009).

### 4.3 Reward‐spatial view neurons in the primate hippocampus

Information about where rewards are located is a key attribute of an episodic memory system. The anterior hippocampus of primates (which corresponds to the ventral hippocampus of rodents) has inputs from brain areas such as the orbitofrontal cortex and amygdala that perform reward processing (Carmichael & Price, 1995; Huang et al., 2021; Pitkanen et al., 2002; Rolls, 2022; Rolls, Deco, et al., 2022d; Rolls, Deco, et al., 2022e; Stefanacci et al., 1996; Suzuki & Amaral, 1994). This connectivity is also proposed to provide information to the human hippocampus about reward location that is needed to provide the goals for navigation (Rolls, 2022; Rolls, Deco, et al., 2022e).

To analyze reward‐related input to the primate hippocampal system, neuronal activity was recorded during a reward‐spatial view association task in monkeys in which one location in each spatial scene on a video monitor, when touched, resulted in a fruit juice reward, and a second location resulted in a less preferred juice reward. The different scenes had different locations for the two reward types (Rolls & Xiang, 2005). Then, 18% of 312 hippocampal cells analyzed responded in different scenes to the location of the preferred reward, and 5% to the location of the less preferred reward (Rolls & Xiang, 2005). Of 44 neurons tested, 60% reversed the location to which they responded when the locations of the preferred rewards were reversed in the scenes, providing evidence that the reward‐place associations could be relearned in a few trials. Most (82%) of the 44 location‐reward neurons in the hippocampus did not respond to object‐reward associations in a visual discrimination task. Thus, the macaque hippocampus represents the reward associations of locations being viewed “out there,” and can store affective information as part of an episodic memory. This provides a way in which the current mood or reward/non‐reward state may influence the retrieval of episodic memories, which is of interest for psychiatric disorders in which sad memories may be emphasized because of altered functional connectivity of the orbitofrontal cortex with hippocampal memory mechanisms (Cheng et al., 2016; Cheng et al., 2018; Rolls, 2016b; Rolls, 2018b; Rolls, 2019b; Rolls et al., 2018; Rolls, Cheng, & Feng, 2020).

There is further evidence that neurons in the primate hippocampus are influenced by rewards. Wirth et al. (2009) described cells that encoded the reward outcome of a trial in an object‐place association task. These findings have been extended by showing that macaque hippocampal neurons reflect outcome information more than prefrontal cortex neurons (Brincat & Miller, 2015). In addition, the reward value of pictures is also represented in the primate hippocampus (Knudsen & Wallis, 2021; Knudsen & Wallis, 2022).

The results indicate that the primate hippocampus can learn associations between viewed locations and objects (Rolls et al., 2005) or rewards (Rolls & Xiang, 2005). Perhaps correspondingly but with a different representation of space, the responsiveness of rodent place cells can be influenced by where rewards are available (Hölscher et al., 2003; Tabuchi et al., 2003). The principle is that the hippocampus may encode information about where emotion‐related (rewarding or punishing) events happened; may be involved in the recall of emotions when particular places are seen later; may provide mechanisms by which the current mood can influence the memories that are recalled; and may be involved via value‐related inputs in whether memories are consolidated (Rolls, 2015; Rolls, 2018b; Rolls, 2022; Rolls, Deco, et al., 2022d; Rolls, Deco, et al., 2022e).

## 5 PATHWAYS BY WHICH SPATIAL VIEW AND RELATED INFORMATION REACHES THE HUMAN HIPPOCAMPAL SYSTEM

**FIGURE 5:**
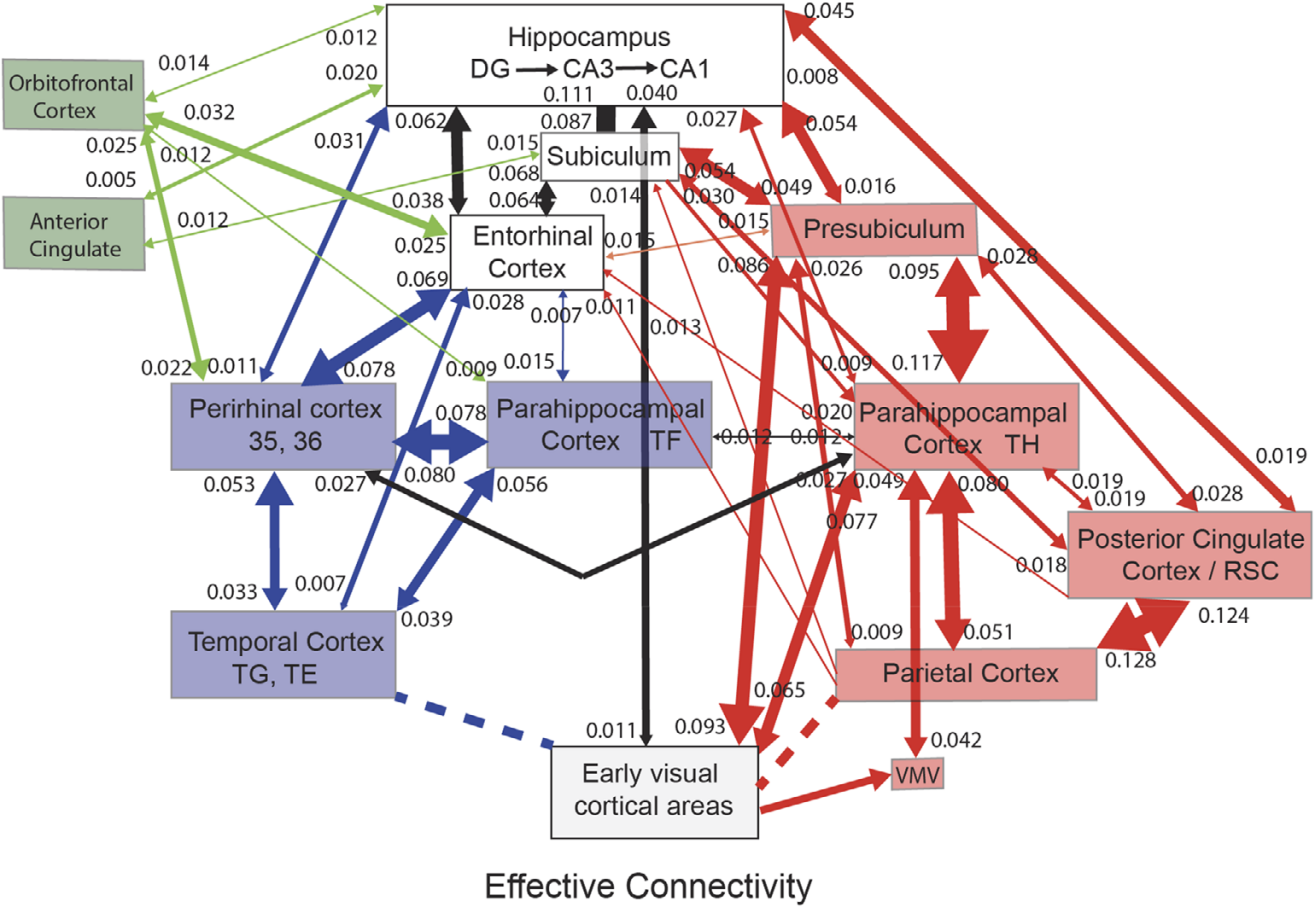
Summary of the effective connectivity of the human hippocampal system measured across all 172 HCP participants at 7T. The maximum value of the effective connectivity is 0.2, and the strength in each direction is shown close to the termination of an arrow. The width of the lines and the size of the arrowheads reflect the strength of the effective connectivity. For areas such as the temporal lobes, the parietal cortex, and the posterior cingulate cortex, there are several subregions in the HCP atlas, and the value of the strongest effectivity connectivity to or from any subarea is shown in this case. Brain regions that are part of the ventral “what” stream are shown in blue, that are part of the dorsal “where” or “action” stream are shown in red, and that involve the orbitofrontal and anterior cingulate cortex reward value stream are in green. The ventromedial visual areas (VMV) and TH include the parahippocampal place/scene area. The early visual areas referred to here include POS1 and ProS. Effective connectivities of less than 0.010 in the stronger direction are not included for clarity. Dashed lines indicate that there are several stages to the connectivity. The summary figure focuses on connectivity of hippocampal system brain regions, and does not show connectivity between other brain systems such as the orbitofrontal cortex and lateral temporal cortex TE and TG. (After Rolls, Deco, et al. (2022d).)

**FIGURE 6:**
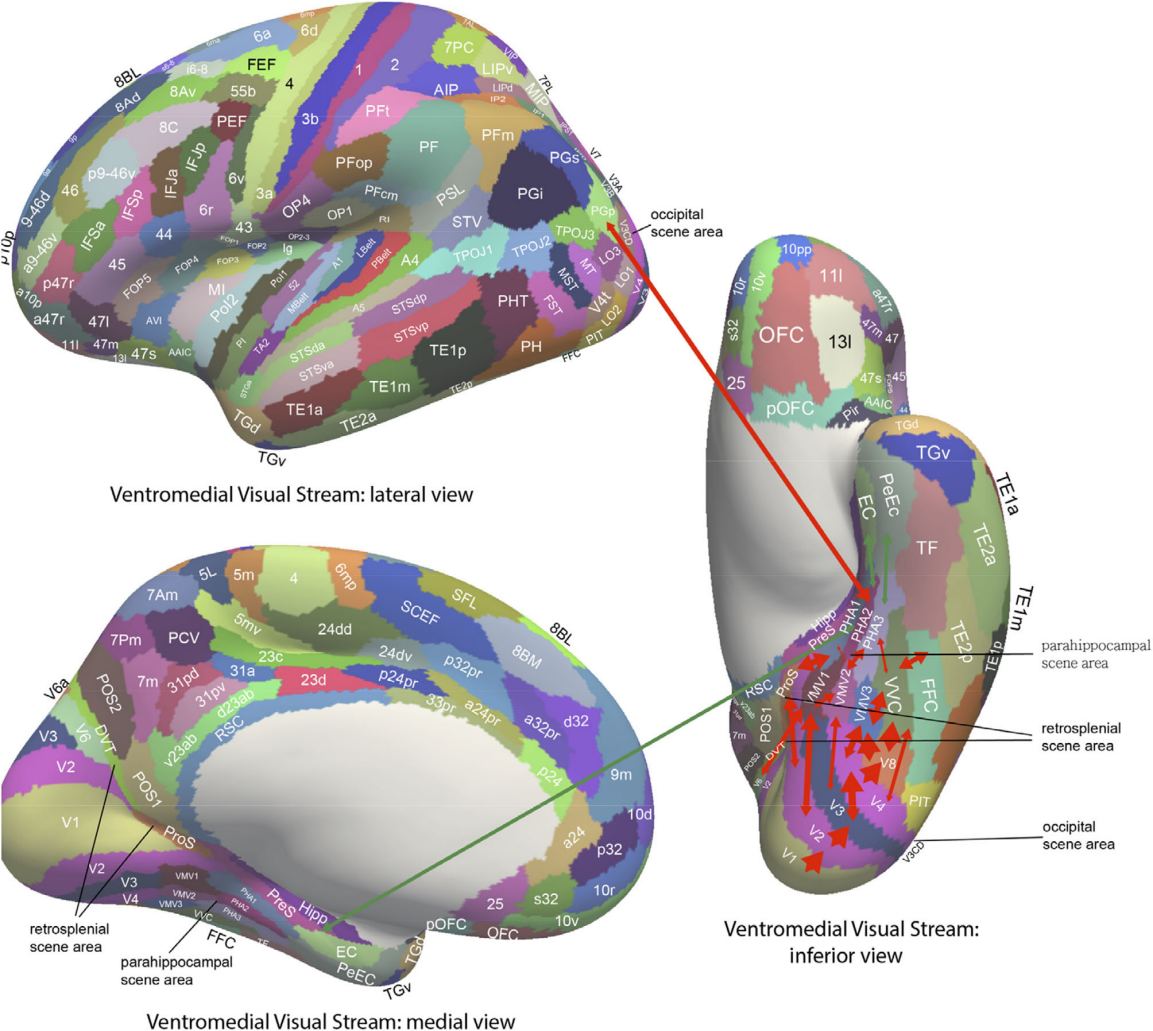
Effective connectivity of the human ventromedial visual cortical stream which reaches the parahippocampal gyrus PHA1–PHA3 regions via ventromedial (VMV) and ventral visual complex (VVC) and ProStriate regions: Schematic overview. Visual scenes are represented in the anterior parts of VMV and the posterior parts of PHA1–PHA3 in what is the parahippocampal scene area (PSA; sometimes called the parahippocampal place area [PPA]) (Sulpizio et al., 2020). The retrosplenial scene area is in a band of cortex in the Prostriate cortex PRoS and dorsal visual transitional (DVT) cortex that is posterior to region RSC (Sulpizio et al., 2020). The occipital scene area is in V3CD and borders V4 (Sulpizio et al., 2020). The green arrows show how the ventromedial visual stream provides “where” input about locations in scenes to the hippocampal memory system from the medial parahippocampal gyrus PHA1–PHA3 region (which corresponds to TH in macaques). The connectivity from PGp to PHA regions is suggested in the text to be involved in idiothetic update of locations in scenes. The widths of the lines and the size of the arrowheads indicate the magnitude and direction of the effective connectivity. (After Rolls, Deco, et al. (2022b).)

**FIGURE 7:**
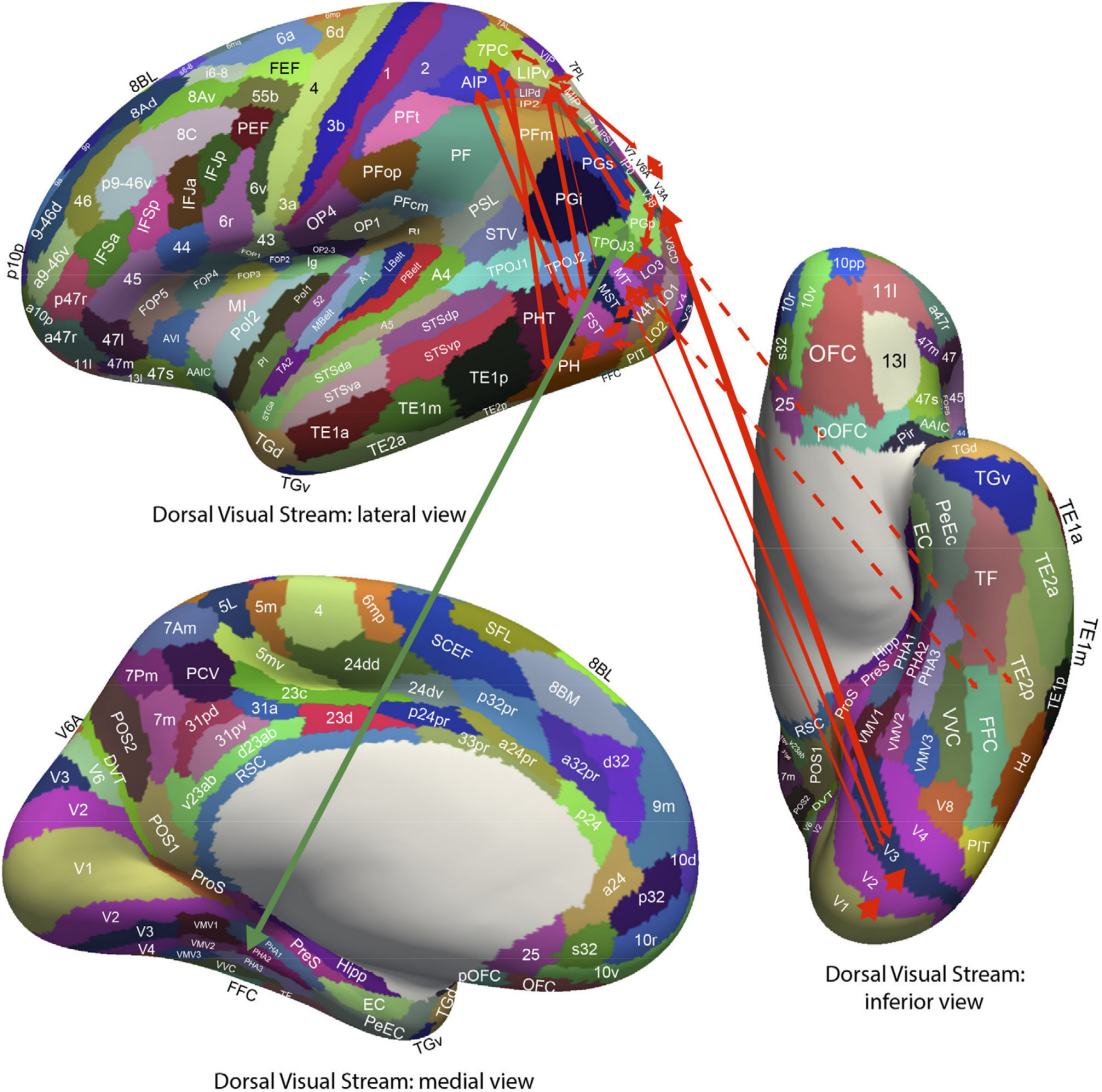
Effective connectivity of the human dorsal visual stream which reaches (partly via V3, V3A, and LO3) the MT+ complex regions (FST, LO1, LO2, LO3, MST, MT, PH, V3CD, and V4t), and then the intraparietal regions (AIP, LIPd, LIPv, MIP, VIP IP0, IP1, and IP2) and then the area 7 regions: Schematic overview. Connectivity to the inferior parietal cortex region PGp, which in turn has effective connectivity to the parahippocampal scene area in PHA1‐3 is shown. Inputs to this stream from ventral stream regions such as FFC and TE2p are shown with dashed lines. (After Rolls, Deco, et al. (2022b).)

**FIGURE 8:**
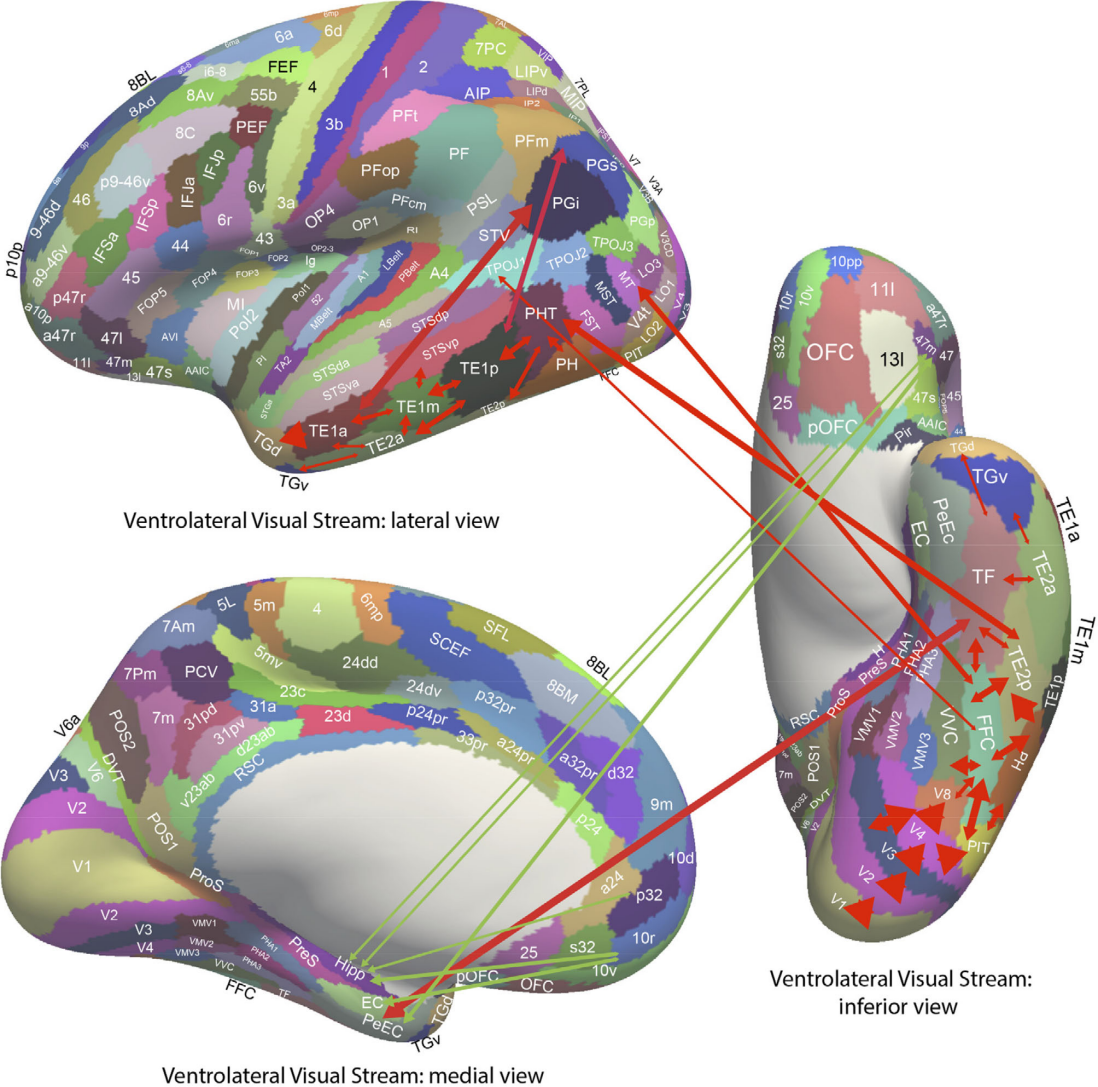
Effective connectivity of the ventrolateral visual stream which reaches inferior temporal cortex TE regions in which objects and faces are represented (red arrows): Schematic overview. One of the red arrows shows how the ventrolateral visual stream provides “what” input to the hippocampal memory system via parahippocampal gyrus TF to perirhinal PeEc connectivity from FFC, PH, TE1p, TE2a, and TE2p. The green arrows show how reward regions of the orbitofrontal cortex; vmPFC (pOFC, 10r, 10v) and pregenual anterior cingulate (a24 and p32); and punishment/non‐reward regions of the lateral orbitofrontal cortex (47 m) have effective connectivity with the hippocampus (Hipp), entorhinal cortex (EC), and perirhinal cortex (PeEC). The ventrolateral visual stream also provides input to the semantic language system via TGd. The ventrolateral visual stream also has connectivity to the inferior parietal visual area PFm, PGs, and PGi as indicated by two green arrows. The widths of the lines and the size of the arrowheads indicate the magnitude and direction of the effective connectivity. (After Rolls, Deco, et al. (2022b).)

**FIGURE 9:**
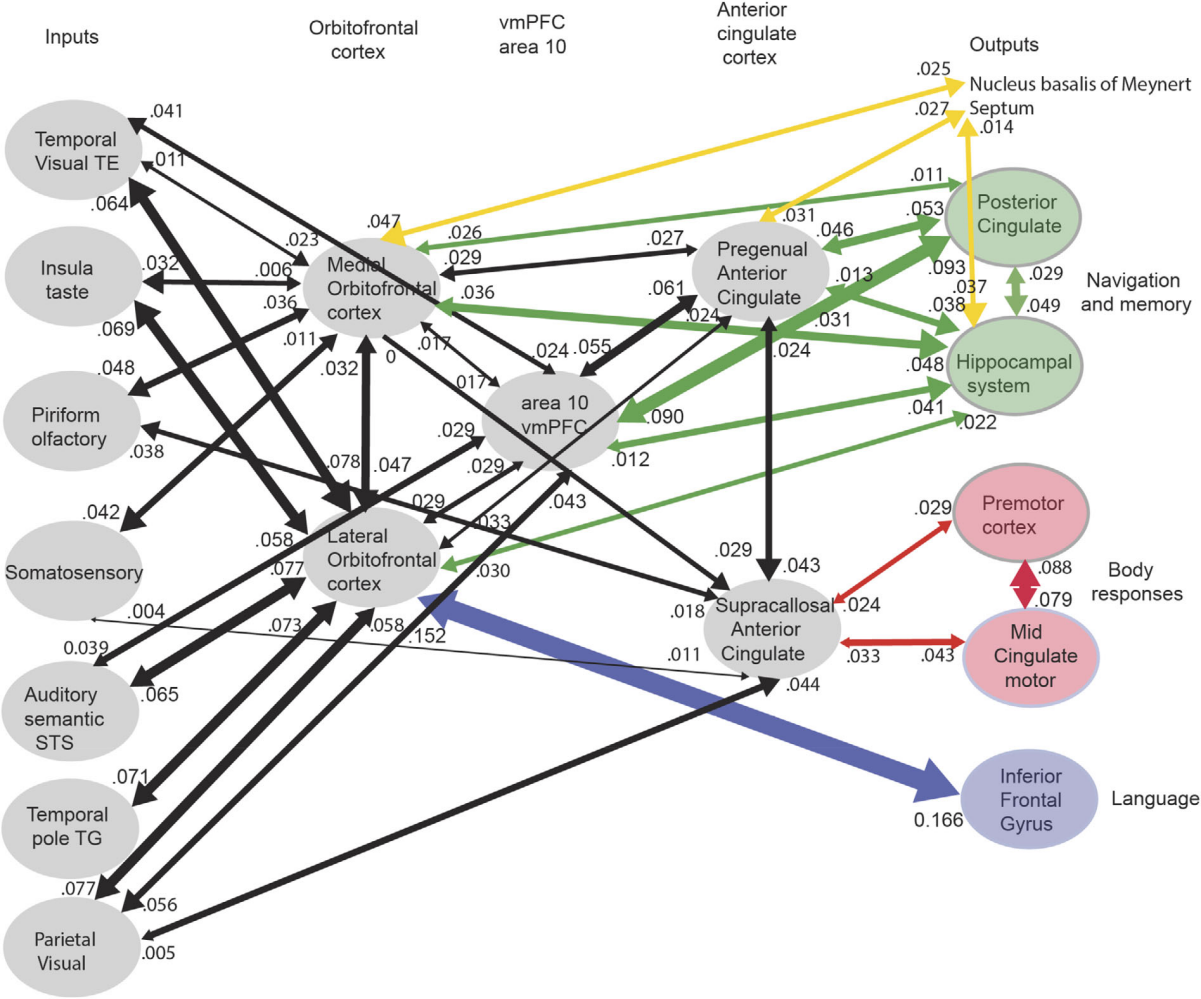
Synthesis of the effective connectivity of the orbitofrontal cortex, ventromedial prefrontal cortex (vmPFC), and anterior cingulate cortex shown in the five central ellipses, with inputs on the left and outputs on the right. The width of the lines is proportional to the effective connectivity in the highest direction, and the size of the arrows reflects the strength of the effective connectivity in each direction. The effective connectivities shown are for the strongest link where more than one link between regions applies for a group of brain regions. Effective connectivities with hippocampal memory system regions are shown in green; with premotor/mid‐cingulate regions in red; with inferior prefrontal language system in blue; and in yellow to the basal forebrain nuclei of Meynert which contains cholinergic neurons that project to the neocortex and to the septal nuclei which contain cholinergic neurons that project to the hippocampus. The somatosensory regions include five and parietal PF and PFop, which also connect to the pregenual anterior cingulate but are not shown for clarity; the parietal regions include visual parietal regions 7, PGi and PFm. The connectivity with dorsolateral prefrontal cortex is not included here for clarity. Connectivity is shown for the five groups in the center of the figure, and does not include, for example, connectivity between somatosensory and premotor cortical regions. (After Rolls, Deco, et al. (2022e).)

To help understand the operation of the human hippocampal memory and navigation system, the connectivity of the human hippocampal system with 360 cortical regions identified multimodally based on anatomy and function in the Human Connectome Project Multimodal Parcellation atlas (HCP‐MMP1) (Glasser, Coalson, et al., 2016) extended to include 66 subcortical areas (Huang et al., 2022) has been investigated (Huang et al., 2021; Ma et al., 2022; Rolls, 2022; Rolls, Deco, et al., 2022c; Rolls, Deco, et al., 2022d; Rolls, Deco, et al., 2022e; Rolls, Wirth, et al., 2022). The connectivity was measured using MRI acquired in more than 170 participants at 7 T in the HCP (Glasser, Smith, et al., 2016) with three different types of measure. Effective connectivity enables the (causal) connectivity in both directions between each pair of brain regions to be measured using, for example, the fMRI BOLD signal. The effective connectivity algorithm that was developed and used measures the functional connectivity between all 360 brain regions at time t and t + tau where tau = 2 s in the BOLD fMRI timeseries (Rolls, Deco, et al., 2022a;Rolls, Deco, et al., 2022b; Rolls, Deco, et al., 2022c; Rolls, Deco, et al., 2022d; Rolls, Deco, et al., 2022e). An effective connectivity matrix is then produced by gradient descent using error correction until the effective connectivity matrix generates computed functional connectivity matrices at time t and t + tau that are close to those measured empirically, using a Hopf bifurcation method and Stuart–Landau oscillators, as fully described and validated elsewhere (Rolls, Deco, et al., 2022a; Rolls, Deco, et al., 2022b; Rolls, Deco, et al., 2022c; Rolls, Deco, et al., 2022d; Rolls, Deco, et al., 2022e; Rolls, Wirth, et al., 2022). The directionality of the effective connectivity that is computed relies on the time delayed version of the functional connectivity matrix. This effective connectivity method was complemented by measurement of functional connectivity between the same brain regions, which given that it is based on Pearson correlations, can provide evidence about interactions between brain regions, but not about the direction or causality of effects (Ma et al., 2022; Rolls, Deco, et al., 2022e). These methods were complemented by diffusion tractography which can measure direct connections between brain regions using completely different methodology not dependent on the BOLD signal, so can provide independent evidence, though not about the direction of connections nor about effects mediated beyond direct connections (Huang et al., 2021; Rolls, Deco, et al., 2022e). Figures 5, 6, 7, 8, 9 summarize some of the results of these investigations (Rolls, Deco, et al., 2022b; Rolls, Deco, et al., 2022c; Rolls, Deco, et al., 2022d; Rolls, Deco, et al., 2022e), and lead to the following points about the connectivity of the human hippocampus and how it relates to spatial view cells.

### 5.1 Spatial scene inputs in humans to hippocampal spatial view neurons

Connectivity is directed to the human hippocampus from a medial posterior part of the parahippocampal gyrus PHA1‐3 (which corresponds to TH in macaques) and the adjoining ventromedial visual areas (VMV1‐3) (Rolls, Deco, et al., 2022b; Rolls, Deco, et al., 2022d) (Figures 5 and 6). This parahippocampal/VMV region (Sulpizio et al., 2020) is a parahippocampal place area (or PSA, PSA, as it responds to viewed scenes not the place where the individual is located) (Epstein, 2005; Epstein, 2008; Epstein & Baker, 2019; Epstein & Julian, 2013; Epstein & Kanwisher, 1998; Kamps et al., 2016; Natu et al., 2021; Sulpizio et al., 2020). Thus, my proposal is that the PSA is a route via which hippocampal spatial view cells receive their information about and selectivity for locations in scenes. This direct route (Rolls, Deco, et al., 2022b; Rolls, Deco, et al., 2022d) is complemented by connectivity via the posterior cingulate cortex (Rolls, Wirth, et al., 2022).

The “ventromedial visual stream” pathway is shown in more detail in Figure 6, which summarizes how in humans visual information reaches the parahippocampal gyrus PHA1–PHA3 regions via ventromedial (VMV1‐3) and ventral visual complex regions (Rolls, Deco, et al., 2022b). In this stream there is effective connectivity from V1 > V2 > V3 > V4. Then V2, V3 and V4 have effective connectivity to the VMV regions, which in turn have effective connectivity to PHA1‐3, which in turn have effective connectivity directed to the hippocampal system (Figure 6, green arrows) (Rolls, Deco, et al., 2022b). In addition, V2 has effective connectivity to the transitional visual areas dorsal transitional visual area (DVT) and the ProStriate (ProS) region, which in humans are where in the HCP‐MMP atlas (Glasser, Coalson, et al., 2016; Huang et al., 2022) the retrosplenial place area is located (Sulpizio et al., 2020); and these regions in turn have effective connectivity to the PHA parahippocampal regions (Figure 6) (Rolls, Deco, et al., 2022b). In humans, the occipital place area OPA is located in V3CD, V3B, and IP0 (Sulpizio et al., 2020).

It is proposed that scene representations are built using combinations of ventral visual stream features that when overlapping in space are locked together by associative learning and can form a continuous attractor network to encode a visual scene (Rolls et al., 2008; Rolls, Deco, et al., 2022c; Rolls & Stringer, 2005; Stringer et al., 2005) using spatial view cells (Georges‐François et al., 1999; Robertson et al., 1998; Rolls, 2022; Rolls et al., 1997b; Rolls et al., 1998; Rolls & Wirth, 2018; Tsitsiklis et al., 2020; Wirth et al., 2017) in the parahippocampal scene (or place) area referred to above, which in turn connects to the hippocampus to provide the “where” component of episodic memory (Rolls, Deco, et al., 2022c; Rolls, Deco, et al., 2022d). This connectivity to the hippocampal scene system is considered further elsewhere (Rolls, 2022; Rolls, Deco, et al., 2022c; Rolls, Deco, et al., 2022d). It is proposed below that a contribution of the dorsal visual stream to scene processing is to provide idiothetic update of spatial view representations.

At first sight, this ventromedial visual cortical stream for “where,” scene, representations, may seem like an unusual proposal. The proposal is that “where” information, about locations in scenes that are encoded by hippocampal spatial view cells, reaches the hippocampus from the PSA in PHA1‐3 and VMV1‐3, which has much connectivity with early ventral visual stream cortical areas. Indeed faces are represented near to the PSA in the fusiform gyrus FFC (Natu et al., 2021; Pitcher et al., 2019; Weiner et al., 2017); ideograms (or logograms) of words are represented just lateral to faces in the visual word form area in the fusiform gyrus (Caffarra et al., 2021; Dehaene et al., 2005; Dehaene & Cohen, 2011; Yeatman & White, 2021); and cortical regions that represent objects are nearby and project forward into the inferior temporal visual cortical areas involved in invariant visual object recognition (Grill‐Spector et al., 2006; Rolls, 2021a; Rolls, 2021f). However, scenes are likely to be represented by spatially contiguous scene features that become associated together in the correct topological arrangement because of the statistics of the inputs (Rolls et al., 2008; Stringer et al., 2005), and thus visual scene representations are likely to be formed from visual features of the type that are represented in ventral stream visual areas.

Highly relevant to these points is that spatial view cells are found in the macaque parahippocampal gyrus as well as the hippocampus (Georges‐François et al., 1999; Robertson et al., 1998; Rolls et al., 1997b; Rolls et al., 1998; Rolls et al., 2005; Rolls & Xiang, 2005). Indeed, it is proposed and likely that many of the neurons in the parahippocampal scene (place) area are spatial view cells, and that this is how scenes are represented in the primate including human brain.

### 5.2 The roles of the parietal cortex in the idiothetic update of hippocampal and parahippocampal spatial view cells and scene representations

What has just been proposed in Section 5.1 raises the question of the role of the parietal cortex, traditionally regarded as the brain region involved in “where” representations (Pitcher & Ungerleider, 2021; Ungerleider & Mishkin, 1982), in the responses of hippocampal (and parahippocampal gyrus) spatial view cells. As shown in Figures 5 and 7, the hippocampal system does receive input from parietal cortex visual regions (Baker, Burks, Briggs, Conner, Glenn, Taylor, et al., 2018; Huang et al., 2021; Ma et al., 2022; Rolls, Deco, et al., 2022b; Rolls, Deco, et al., 2022c; Rolls, Deco, et al., 2022d). Inputs are also received via the visuomotor parts of the posterior cingulate cortex (Baker, Burks, Briggs, Conner, Glenn, Manohar, et al., 2018; Rolls, Wirth, et al., 2022).

In more detail, the effective connectivity of the human dorsal visual cortical stream is shown in Figure 7 (Rolls, Deco, et al., 2022b). There is effective connectivity from parietal cortex regions to the PSA, as illustrated in Figure 7. Strong effective connectivity is directed from inferior parietal region PGp to the PSAs in PHA1‐3 (Figure 7) (Rolls, Deco, et al., 2022c). PGp receives its inputs from parietal area 7 regions and intraparietal regions (Figure 7) (Rolls, Deco, et al., 2022b) involved in visual motion analysis and in coordinate transforms from retinal to head‐based and then to world‐based (allocentric) coordinates (Rolls, 2020; Salinas & Sejnowski, 2001; Snyder et al., 1998). These coordinate transforms are fundamental for self‐motion update of scene representations, so that the spatial view neurons in the PSA can represent where in a scene the individual is looking independently of eye position, head direction, and even the place of the head in the environment, when the view details are obscured or in the dark (Robertson et al., 1998; Rolls, 2020). Given these two lines of evidence, it is proposed that the parietal cortex has the role of idiothetic update of the scene representations in the PSA and thereby in the hippocampus (Rolls, Deco, et al., 2022b; Rolls, Deco, et al., 2022c). Thus, the hypothesis is that the “where” scene representations in the human ventromedial visual stream are built by combinations of ventral stream spatial features, and the viewed position in the scene is idiothetically updated by coordinate transforms to the allocentric level of scenes (Rolls, 2020) by the parietal cortex inputs to the PSA (Rolls, Deco, et al., 2022b; Rolls, Deco, et al., 2022c). Optic flow is a signal that can be used in idiothetic update, and in addition it is known that optic flow regions such as V3, V6 and the MT+ complex have functional (Rolls, Deco, et al., 2022b; Sherrill et al., 2015) and effective (Rolls, Deco, et al., 2022b) connectivity with regions involved in navigation such as the hippocampus, retrosplenial cortex, and posterior parietal cortex, and that optic flow activates regions that are close to cortical scene regions in the occipital, retrosplenial, and parahippocampal scene regions (Sulpizio et al., 2020).

It is emphasized in this approach that the “path integration” that is required for idiothetic update in humans and other primates involves eye position as well as head direction and the place where the individual is located, and much takes place in the dorsal visual system regions in the cortex in the intraparietal sulcus and area 7 (Rolls, 2020; Rolls, Deco, et al., 2022c). The mechanisms by which these coordinate transforms are implemented in the parietal cortex (Rolls, 2020) are considered in Section 6. A real problem with hypothesizing that path integration of any type occurs within the hippocampus is that the energy landscape of any continuous attractor network representation of place or spatial view in the hippocampus that utilized idiothetic update (Rolls & Stringer, 2005; Stringer et al., 2002; Stringer et al., 2005) would be so distorted by association with the “what” and reward information used for episodic memory that it would be very poor at path integration, as the energy landscape would be too bumpy because of the associations (cf. Spalla et al., 2021).

What I am proposing is that the primate including human hippocampal “where” system has two components or parts. One is a ventromedial visual cortical stream scene system with spatial view cells formed by visual feature components locked together in the correct spatial arrangement using overlapping receptive fields and associatively modified synapses of recurrent collaterals of the pyramidal cells that learn from the statistics of the neuronal activity how close the features are in the scene (Rolls et al., 2008; Stringer et al., 2005). This is located in ventral visual stream areas such as the ventromedial visual areas VMV1‐3 and the PSA (Rolls, Deco, et al., 2022b; Rolls, Deco, et al., 2022d). The second component is the dorsal visual stream areas extending into the intraparietal sulcus areas such as LIP, VIP and MIP, area 7, and PGp (in the HCP‐MMP atlas (Glasser, Coalson, et al., 2016; Huang et al., 2022)), which are involved in idiothetic update of spatial view cells by computing representations of allocentric view that are invariant with respect to retinal position, eye position, head direction, and the place where the individual is located (Rolls, 2020; Rolls, Deco, et al., 2022b; Rolls, Deco, et al., 2022c). The connectivity from the hippocampus and parahippocampal gyrus to the parietal cortex may further provide a route for allocentric locations in space with associations with rewards, goals, or objects and remembered using hippocampal mechanisms to produce navigation and visuomotor actions in space that require transforms to egocentric coordinates (Rolls, Deco, et al., 2022c).

### 5.3 Ventrolateral visual stream “what” inputs to the human hippocampal system

To add to the recently developing understanding of human hippocampal system inputs, Figure 8 shows the ventrolateral visual stream in humans progressing via V1 > V2 > V4 > FFC (which contains representations of faces, objects, and even words in the visual word form area laterally) > the anterior temporal lobe TE regions (Rolls, Deco, et al., 2022b) where invariant representations of objects and faces are built (Rolls, 2000; Rolls, 2021a; Rolls, 2021f). This pathway provides “what” inputs to the hippocampal memory system via parahippocampal area TF, which is lateral and anterior to the scene area in PHA1‐3 (Figure 8) (Rolls, Deco, et al., 2022b).

Figure 8 also illustrates some of the reward‐related inputs to the human hippocampal system with green arrows, and these are considered next.

### 5.4 Reward value/emotion‐related inputs to the human hippocampus

**FIGURE 10:**
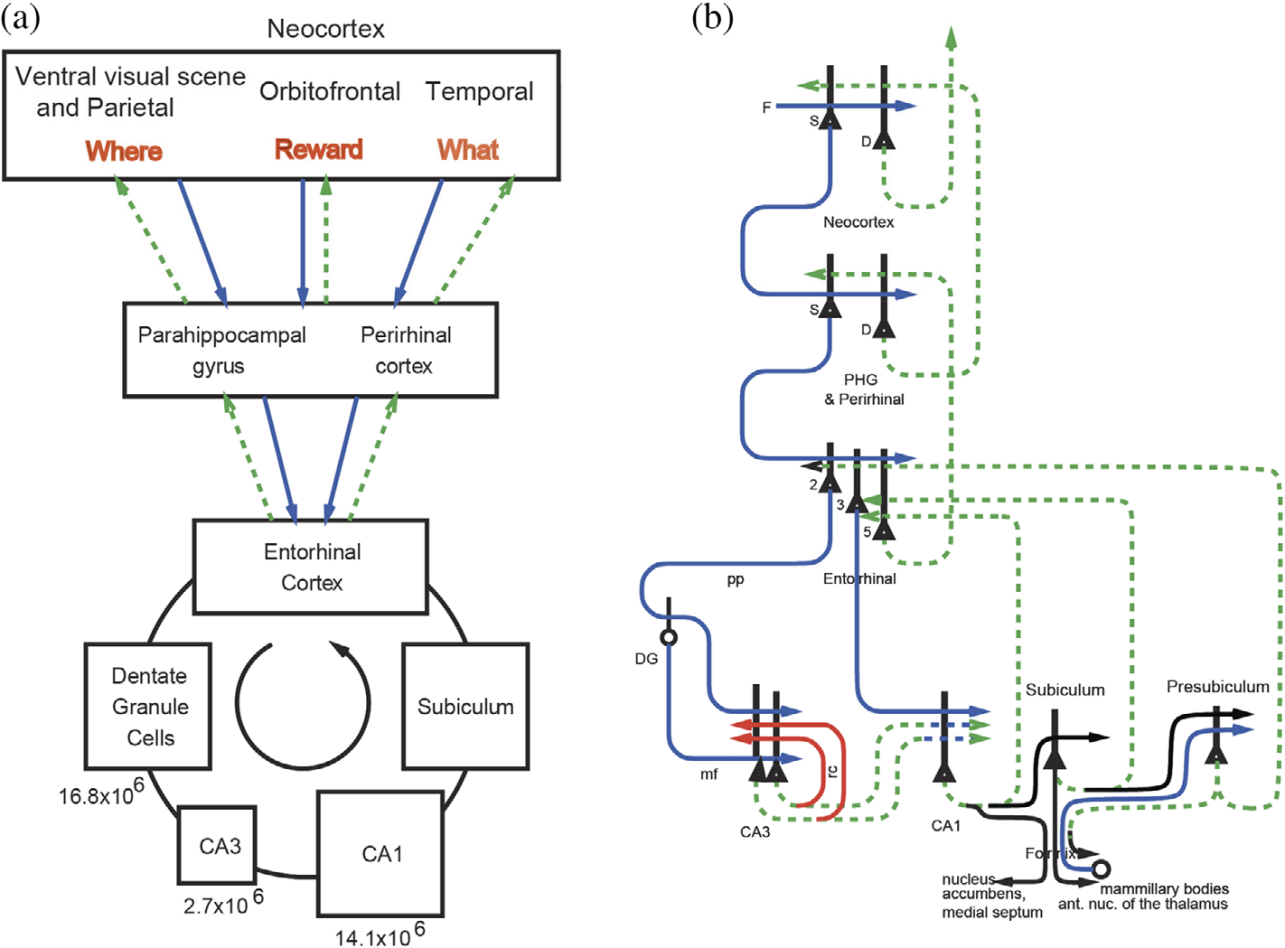
The human/ primate hippocampus receives neocortical input connections (blue) not only from the “what” temporal lobe and “where” medial temporal lobe scene and parietal lobe systems, but also from the “reward” prefrontal cortex areas (orbitofrontal cortex, ventromedial prefrontal cortex [vmPFC], and anterior cingulate cortex) for episodic memory storage; and has return backprojections (green) to the same neocortical areas for memory recall. There is great convergence via the parahippocampal gyrus, perirhinal cortex, and dentate gyrus in the forward connections down to the single network implemented in the CA3 pyramidal cells, which have a highly developed recurrent collateral system (red) to implement an attractor episodic memory by associating the what, where and reward components of an episodic memory. (a) Block diagram. (b) Some of the principal excitatory neurons and their connections in the pathways. Time and temporal order are also important in episodic memory, and may be computed in the entorhinal–hippocampal circuitry (Rolls & Mills, 2019). D: deep pyramidal cells. DG: dentate granule cells. F: forward inputs to areas of the association cortex from preceding cortical areas in the hierarchy. mf: mossy fibers. PHG: parahippocampal gyrus and perirhinal cortex. pp: perforant path. rc: recurrent collateral of the CA3 hippocampal pyramidal cells. S: superficial pyramidal cells. 2: pyramidal cells in layer 2 of the entorhinal cortex. 3: pyramidal cells in layer 3 of the entorhinal cortex. The thick lines above the cell bodies represent the dendrites. The numbers of neurons in different parts of the hippocampal trisynaptic circuit in humans (Rogers Flattery et al., 2020) are shown in (a), and indicate very many dentate granule cells, consistent with expansion encoding and the production of sparse uncorrelated representations prior to CA3 (Rolls, 2016c; Rolls, 2021d)

First, reward value and emotion‐related inputs reach the human hippocampal system from the orbitofrontal cortex and anterior cingulate cortex partly via the perirhinal and entorhinal cortex (Figures 5, 8, and 9) (Ma et al., 2022; Rolls, Deco, et al., 2022b; Rolls, Deco, et al., 2022d; Rolls, Deco, et al., 2022e), and perhaps even more directly (Huang et al., 2021). This important route for reward to gain access to the hippocampus in addition to “what” and “where” inputs is shown in the updated hippocampal schematic connection diagram in Figure 10, which shows how reward value information could be associated with “where” spatial view cell activity in hippocampal CA3, if not before.

The connectivity for these reward value and emotion‐related inputs to reach the human hippocampus is shown in more detail in Figures 8 and 9 (Rolls, Deco, et al., 2022b; Rolls, Deco, et al., 2022e). The sensory/perceptual cortical regions on the left of Figure 9 provide visual, taste, olfactory, and auditory input not coded in terms of their reward value to the orbitofrontal cortex (Rolls, 2019a; Rolls, 2019b; Rolls, 2021a). The representation of these signals in the orbitofrontal cortex is in terms of their reward value, as shown by stimulus–reward learning and reversal; and by satiation which selectively reduces the reward value (Berlin et al., 2004; Grabenhorst & Rolls, 2011; Hornak et al., 1996; Hornak et al., 2003; Hornak et al., 2004; Rolls, 2019b; Rolls, 2021e; Rolls et al., 1994; Rolls, Cheng, & Feng, 2020; Rolls, Vatansever, et al., 2020; Thorpe et al., 1983). Reward is represented especially in the human medial orbitofrontal cortex, and punishment and non‐reward in the lateral orbitofrontal cortex (Grabenhorst & Rolls, 2011; Rolls, Cheng, & Feng, 2020). The medial and lateral orbitofrontal cortex have effective connectivity to the pregenual anterior cingulate cortex, in part via the ventromedial prefrontal cortex (vmPFC) (Figure 9) (Rolls, Deco, et al., 2022e). The medial and lateral orbitofrontal cortex, and the vmPFC, then have effective connectivity with the hippocampal system (perirhinal and entorhinal cortex, and perhaps directly with the hippocampus) (Figures 8 and 9) (Rolls, Deco, et al., 2022e). The memory‐related part of the posterior cingulate cortex provides additional connectivity between these reward‐related regions and the hippocampal system (Figure 5) (Rolls, Deco, et al., 2022d; Rolls, Wirth, et al., 2022).

These neural connectivity investigations provide clear evidence on how reward value and emotion‐related information can reach the human hippocampal system. It is proposed that in the hippocampus, including in CA3, reward value information can be associated with spatial view information to enable the reward and emotional aspects of episodic memory to become part of the episodic memory (Rolls, 2022; Rolls, Deco, et al., 2022e). The return pathways from the hippocampus to the orbitofrontal cortex, pregenual anterior cingulate cortex, and vmPFC shown in Figures 5, and 8, 9, 10 provide a route for the reward and emotional value of an episodic memory to be recalled back from the hippocampus to the orbitofrontal and anterior cingulate cortex and vmPFC (Rolls, 1995; Rolls, 2021a; Rolls & Treves, 1994; Treves & Rolls, 1994), from which it can influence other brain regions involved in actions (Rolls, 2021a; Rolls, Cheng, & Feng, 2020; Rolls, Deco, et al., 2022e).

This connectivity described in humans also shows how a key component of navigation, the goal or reward toward which the navigation is directed, reaches the human hippocampal system, where navigation may be guided by a remembered sequence of spatial view locations encoded by spatial view cells (Rolls, 2021a; Rolls, 2021b; Rolls, Deco, et al., 2022e).

In addition, the orbitofrontal cortex and pregenual anterior cingulate cortex have connectivity (yellow in Figure 6) directed to the human cholinergic basal nucleus of Meynert which projects to the neocortex, and septal region which projects to the hippocampus, and these pathways are proposed to influence memory consolidation including consolidation into long‐term semantic memory (Rolls, 2022; Rolls, Deco, et al., 2022e).

## 6 MECHANISMS FOR SPATIAL COORDINATE TRANSFORMS USED IN THE IDIOTHETIC UPDATE OF SPATIAL VIEW NEURONS

Gain modulation to produce coordinate transforms is a well‐established principle of operation of neuronal systems in the dorsal visual system (Salinas & Abbott, 1995; Salinas & Abbott, 1996;Salinas & Abbott, 2001; Salinas & Sejnowski, 2001). The term gain field describes the finding that the response of a neuron in parietal areas 7a, LIP, and VIP to a visual stimulus at a given position on the retina (the neuron's receptive field) can be modulated (decreased or increased) by a modulating factor, eye position (the angle of the eye in the head) (Andersen, 1989; Andersen et al., 1985; Andersen & Mountcastle, 1983; Duhamel et al., 1997). Each neuron thus responds best to a combination of retinal and eye position. The gain modulation by eye position occurs in a spatially systematic and nonlinear way such that the output of the population of neurons encodes the position of the stimulus relative to the head, by taking into account both retinal position and eye position (Salinas & Abbott, 2001; Salinas & Sejnowski, 2001). This gain modulation can be thought of as shifting the retinal receptive field of the population of neurons so that they represent direction relative to the head, which is a spatial coordinate transform.

This gain‐modulation principle was developed to produce a model of the coordinate transforms that take place in the dorsal visual system in parietal cortex regions, in areas such as LIP, VIP, and area 7, to show how idiothetic update of spatial view cells can be produced (Rolls, 2020).

The first stage is similar in principle to that described previously (Salinas & Abbott, 2001; Salinas & Sejnowski, 2001), and transforms from retinal to head‐based coordinates using gain modulation of the position of the stimulus on the retina by eye position (Rolls, 2020). However, a key development of the gain modulation mechanism modeled is that it includes slow learning in which the synaptic modification includes a trace of recent neuronal activity (Rolls, 2020). This enables the system to benefit from the fact that the position in e.g. head‐based space may be constant in real environments over a series of eye position and retinal position values (Rolls, 2020), and is analogous to the same process that is proposed to learn transform invariances of objects in the ventral visual system (Rolls, 2021f). Neurons that respond in head centered coordinates are found in macaque areas VIP and LIP (Andersen, 1989; Andersen et al., 1985; Andersen & Mountcastle, 1983; Duhamel et al., 1997). The second and third stages are new proposals (Rolls, 2020).

**FIGURE 11:**
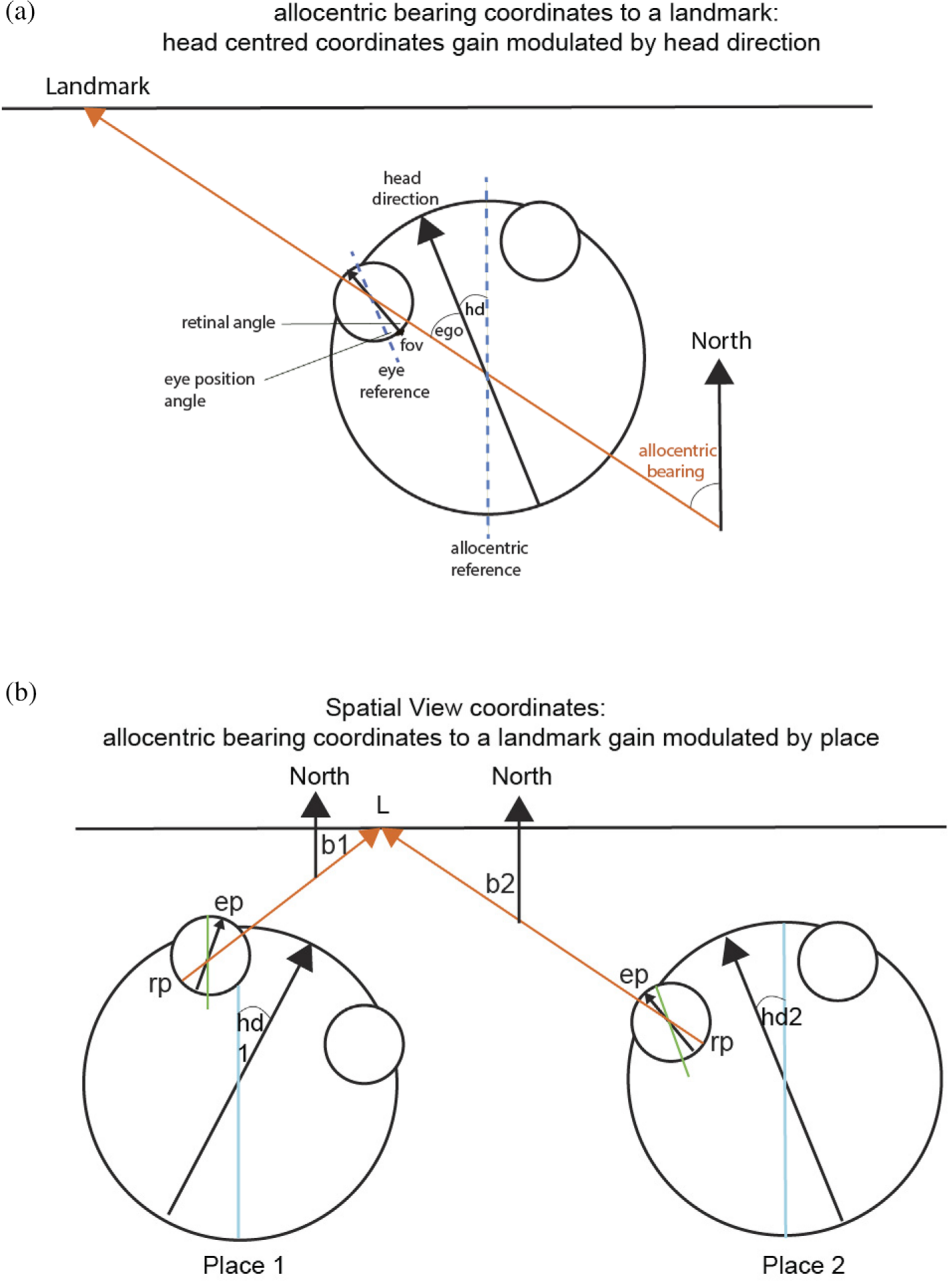
(a) Gain modulation by head direction (hd) to produce a representation in allocentric bearing coordinates (relative to north) to a location in space at which there is a landmark. The head direction is the angle between north (indicated by the long blue line) and the direction of the head (indicated by the long black arrow). A bearing coordinate to a landmark L is represented by all combinations of head direction, eye position (ep), and retinal position that correspond to a given bearing from the individual to a landmark in allocentric space indicated by the line with a red arrow. The large circle is the head, and the two small circles are the eyes. The allocentric bearing to the landmark L is given by the angle between north and the red line from the individual (observer) to the landmark. In this case, the allocentric reference frame (indicated by the blue dashed line) is aligned with north, but it could be specified by dominant environmental cues in a particular environment. The large black arrow labeled “head direction” specifies the direction relative to the allocentric reference framework in which the head is facing, with the head direction angle “hd” as shown. The head direction (hd) is thus in allocentric coordinates. The egocentric bearing to a landmark (“ego”) is the angle between the head direction and the line of sight to the landmark. (As the diagram makes clear, combining the egocentric bearing of the landmark and the head direction yields the allocentric bearing to a landmark.) The diagram also shows how the eye position (the angle between the eye reference frame which is aligned with the head direction as shown), and the retinal angle (the angle between the fovea [“fov”] and the place on the retina of the image of the landmark) are relevant. (b) Gain modulation by place of a bearing representation from the previous stage can produce a representation of a landmark L in a scene in allocentric spatial view coordinates. This signal could be used to idiothetically update spatial view cells. b1: bearing of the landmark from place 1; b2: bearing of the landmark from place 2; hd1: head direction 1; hd2: head direction 2; ep: eye position; rp: retinal position. A landmark L at a location being viewed in allocentric space, that is, a spatial view, is represented by transforms over all places, building on transforms over head direction learned in the previous stage, and transforms over eye position learned in the layer before that. Other conventions as in Figure 8a. (Modified from Rolls (2020).)

In a second stage, using the same gain modulation mechanism combined with slow learning, gain modulation by head direction is used to transform head‐based coordinates into allocentric bearing to a landmark (compass direction) coordinates, as illustrated in Figure 11a (Rolls, 2020). Neurons of this type encoding direction in world‐based coordinates have been found in area 7 (Snyder et al., 1998), and in a region to which it projects, the posterior cingulate cortex (Dean & Platt, 2006).

In a third stage, using the same gain modulation mechanism combined with slow learning, gain modulation by place is used to transform allocentric bearing to a landmark neurons into allocentric spatial view neurons, as illustrated in Figure 11b (Rolls, 2020). Spatial view neurons are found in the parahippocampal gyrus and hippocampus, and their idiothetic update may be implemented in parietal areas such as PGp, which receives from area 7 and intraparietal regions, and which projects to the hippocampal system directly (Rolls, Deco, et al., 2022b; Rolls, Deco, et al., 2022c; Rolls, Deco, et al., 2022d) and via the posterior cingulate cortex (Rolls, Wirth, et al., 2022).

Approaches to coordinate transforms based on research in rodents have been described elsewhere (Bicanski & Burgess, 2018; Byrne et al., 2007).

## 7 ROLE OF HIPPOCAMPAL SPATIAL VIEW NEURONS IN NAVIGATION

It has been shown above that hippocampal spatial view neurons may be important in episodic memory, for which they provide the “where” component. But spatial view neurons may also be useful in navigation in primates including humans (Rolls, 2021b).

Navigation using an internal map of space with places in the map organized to reflect the topology of the space has been a fruitful field of enquiry in neuroscience inspired by the book “The Hippocampus as a Cognitive Map” (O'Keefe & Nadel, 1978), and is supported by the discovery of place cells in the hippocampus of the rat (O'Keefe & Dostrovsky, 1971) and macaque (Rolls & O'Mara, 1995), and grid cells in the entorhinal cortex (Fyhn et al., 2004) of the rat. Schemes have been devised about how this internal map of places in the world and their relative positions can be used with head direction cells and path integration to account for navigation in what are complicated computations (Bicanski & Burgess, 2018; Edvardsen et al., 2020; Hartley et al., 2014).

But is that how humans generally navigate? It has been argued that with the great development of the primate visual system, navigational strategies frequently make use of the visual inputs to navigate using distant visual landmarks (Rolls, 2021b), and this appears to be characteristic of humans (Waller & Lippa, 2007). In contrast, in rodents navigation may be more based on the place where the rodent is located, with olfactory and somatosensory cues of importance in specifying the place where the rodent is currently located, during navigation which may frequently be in the dark.

In this context, a new theory has been proposed of mechanisms of navigation in primates including humans in which spatial view cells found in the primate hippocampus and parahippocampal gyrus are used to guide the individual from landmark to landmark (Rolls, 2021b). The navigation involves approach to each landmark in turn (taxis), using spatial view cells to identify the next landmark in the sequence, and does not require a topological map (Rolls, 2021b). Two other cell types found in primates, whole body motion cells, and head direction cells, can be utilized in the spatial view cell navigational mechanism, but are not essential. If the landmarks become obscured, then the spatial view representations can be updated by self‐motion (idiothetic) path integration using spatial coordinate transform mechanisms in the primate dorsal visual system to transform from egocentric to allocentric spatial view coordinates (Rolls, 2020). A continuous attractor network or time cells or working memory is used in this approach to navigation to encode and recall the spatial view sequences involved (Rolls, 2021b). The theory has been made explicit in models of navigation, which are illustrated by computer simulations (Rolls, 2021b). It is proposed that a navigational strategy utilizing spatial view cells is used frequently in humans, and is relatively simple because primates have spatial view neurons that respond allocentrically to locations in spatial scenes (Rolls, 2021b). Consistent with this neuronal level theory for navigation in primates including humans, there is evidence in humans that the parahippocampal place (or scene) area is critical for landmark recognition (Epstein & Vass, 2014).

## 8 MECHANISMS FOR THE RECALL OF SPATIAL SCENE INFORMATION FROM THE HIPPOCAMPUS TO THE NEOCORTEX

A standard theory of the operation of the hippocampus for episodic memory is that “what” and “where” inputs are associated together in an autoassociation or attractor network in hippocampal CA3, and that the whole memory can then be retrieved in CA3 from either part in the process of completion (McClelland et al., 1995; McNaughton & Morris, 1987; Rolls, 1987; Rolls, 1989b; Rolls, 2018a; Rolls, 2021a; Rolls & Treves, 1994; Treves & Rolls, 1994). However, if the recalled hippocampal memory is to be used, it must be retrieved from the hippocampus. The theory of the retrieval of information from the hippocampus that was developed (Rolls, 1989b) and then specified quantitatively (Treves & Rolls, 1994) is that the backprojection pathways from the hippocampus to the neocortex shown with green dashed lines in Figure 10 are used for the memory recall. The theory is that during the original learning of the episodic memory, the backprojection pathways from the hippocampus to the neocortex are active, and allow the active backprojection neurons to be associated using pattern association with whatever neocortical neurons are active during the formation of the memory (Rolls, 1989b; Rolls & Treves, 1994; Treves & Rolls, 1994). The quantitative analysis shows that the number of memories *p* that can be recalled to the neocortex is

(1) $$p≅\frac{C}{a\ln\left(1/a\right)}k$$

where *C* is the number of associatively modifiable backprojection synapses onto each neocortical pyramidal cell, *a* is the sparseness of the representation in the backprojection pathways, and *k* is a factor that depends weakly on the detailed structure of the rate distribution, on the connectivity pattern, and so forth, but is roughly in the order of 0.2–0.3 (Treves & Rolls, 1994). [The sparseness *a* in this equation is strictly the population sparseness (Franco et al., 2007; Treves & Rolls, 1991). The population sparseness *a* ^p^ would be measured by measuring the distribution of firing rates of all neurons to a single stimulus at a single time.] This remains the only quantitative theory of the recall of information from the hippocampus to the neocortex, and the only quantitative theory of why in the neocortex there are as many backprojection synaptic connections onto each neuron as forward connections, and for that matter why there are as many backprojection connections onto each neocortical pyramidal cell as there are connections onto each CA3 cell, which is >10,000 (Rolls, 2016a; Rolls, 2021a; Treves & Rolls, 1994).

In relation to spatial view cells, it is this mechanism that allows, for example, the spatial view that is associated with an object or reward in the hippocampus in episodic memory to be later recalled to the neocortex, where the recalled allocentric spatial view can be used by neocortical mechanisms at least partly in the parietal lobe to perform visuomotor actions to obtain the object or reward at that allocentric location in space. The actions might include locomotion and navigation to the correct spatial location, and visuomotor actions such as reaching out to that location to grasp the object or reward (Andersen, 1995; Andersen et al., 2000; Bisley & Goldberg, 2010; Gnadt & Andersen, 1988; Orban et al., 2021; Passarelli et al., 2021; Rolls, 2021a; Rolls, 2021b; Rolls, 2022; Rolls, Deco, et al., 2022c). The theory of recall using backprojections can be extended to the case where spatial information is structured into charts or cognitive maps, for the theory of recall to the neocortex via a set of heteroassociative backprojection connections (Figure 10) (Treves & Rolls, 1994) follows the same approach for charts as the theory that applies to an attractor network (Battaglia & Treves, 1998).

**FIGURE 12:**
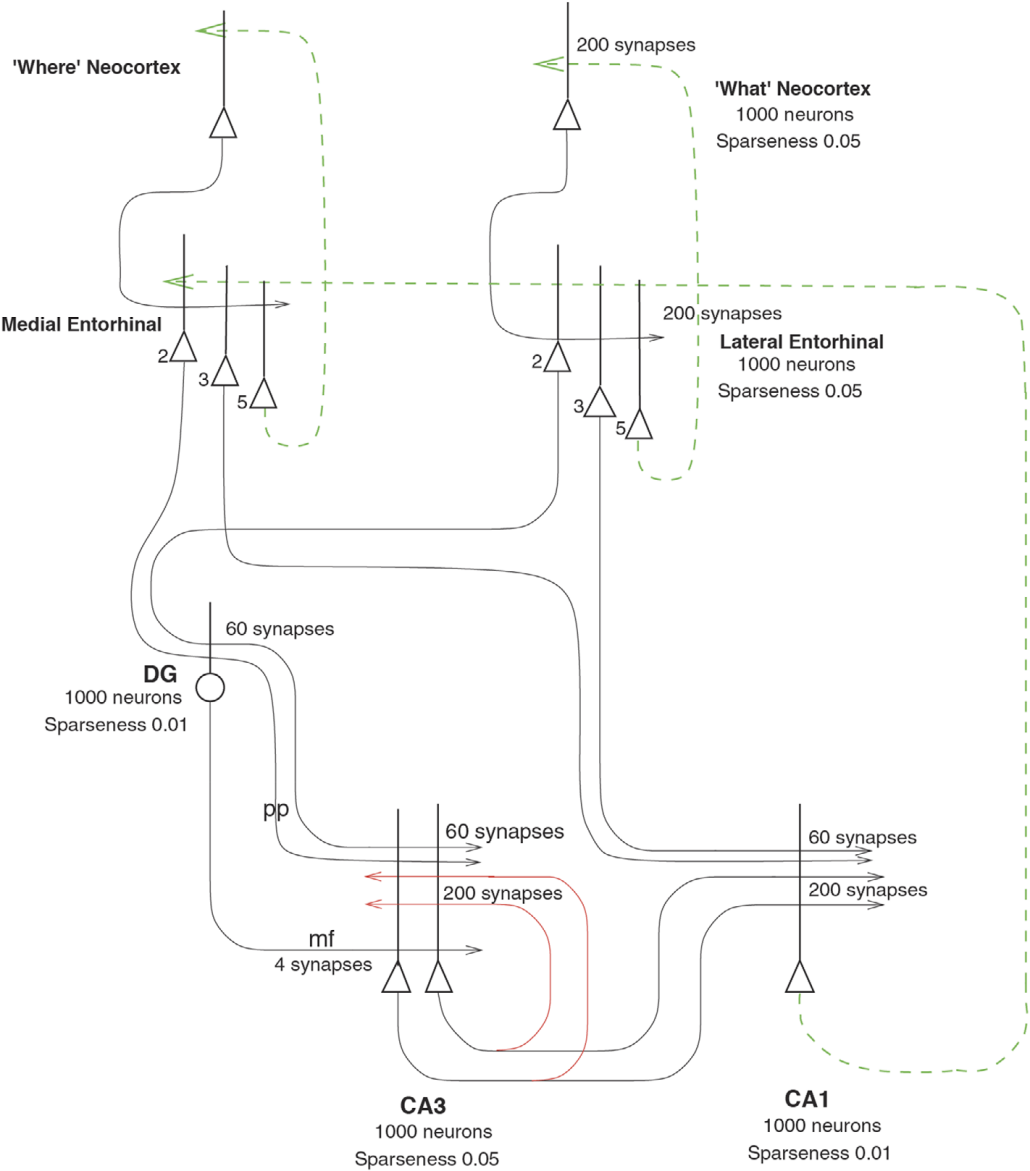
Simulation of neocortical “what” and “where” inputs to the hippocampus for the storage of episodic memory, and for the recall of “what” (object) and “where” (spatial view) information back to the “what” and “where” neocortex. The pyramidal cells bodies are shown as triangles, the dendrites as the thick lines above the cell bodies, and the axons as thin lines terminated with an arrow. The backprojection pathways for memory recall are shown in dashed green lines, and in red the CA3 recurrent collaterals via which “what” and “where” representations present at the same time can be associated during episodic memory storage, and via which completion of a whole memory from a part can occur during recall. All synapses are associatively modifiable except for the dentate gyrus (DG) mossy fiber (mf) synapses on the CA3 pyramidal cells. The dentate granule cells and the CA1 cells operate as competitive networks

The theory of recall of information from the hippocampus as far as the entorhinal cortex was tested in a simulation of the hippocampal system including the entorhinal cortex, dentate gyrus, CA3, and CA1 (Rolls, 1995) (cf. Hasselmo & Wyble, 1997). That research has now been extended to include also a “where” neocortical representation, a “what” neocortical representation, a “where” entorhinal representation in the medial entorhinal cortex (posterior in primates [Ohara et al., 2021]), and a “what” entorhinal representation in the lateral entorhinal cortex (anterior in primates (Ohara et al., 2021)) (Figure 12). The recall of information to the “where” neocortical region corresponding to, for example, the parietal cortex of, for example, spatial view information operates correctly with the expected high capacity in this first simulation of the recall process from the hippocampus all the way back to neocortical regions (Figure 12). The details of the simulation are similar to those described earlier (Rolls, 1995), with associative synaptic modification throughout except for the dentate connections via the mossy fibers to the CA3 cells. During learning, these mossy fiber synapses force new sets of CA3 neurons to fire for each episodic memory in a pattern separation effect, but during recall the entorhinal cortex to CA3 synapses are important, because they are associatively modifiable, and because they are large in number (Treves & Rolls, 1992). The entorhinal cortex, dentate granule cells, and CA1 cells operate as competitive networks to help categorization, as described in detail elsewhere where MATLAB code is made available (Rolls, 2016a; Rolls, 2021a).

## 9 A THEORY AND MODEL OF THE FORMATION OF PRIMATE SPATIAL VIEW CELLS RELATED TO FOVEAL VISION, AND OF RODENT PLACE CELLS RELATED TO A WIDE FIELD OF VIEW

Primates, including humans, have a highly developed fovea, and visual cortical areas for object recognition for what is at the fovea, and an eye movement control system for foveation, and can explore, and remember, what is present at places “out there” in the environment without needing to visit those places. Spatial view cells in primates, given the evidence described here, are likely to be of fundamental use in a primate memory system, by providing a representation of a part of space that would not depend on exactly where the monkey or human was located, and that could be associated with objects or rewards present in those viewed spatial locations. This would enable humans, for example, to remember the viewed location where a person had been seen. These primate spatial representations would also be useful in remembering trajectories through gazing at landmarks, of use, for example, in spatial navigation (Rolls, 2021b; Rolls & Wirth, 2018; Wirth et al., 2017).

The spatial representation in the rodent hippocampus, of the place where the rodent is, may be related to their large visual field of view compared to the primate, and absence of foveate vision and eye movements to fixate distant locations in scenes. A hypothesis on how this difference could be produced by a similar computational process in rodents and primates is as follows (de Araujo et al., 2001).

**FIGURE 13:**
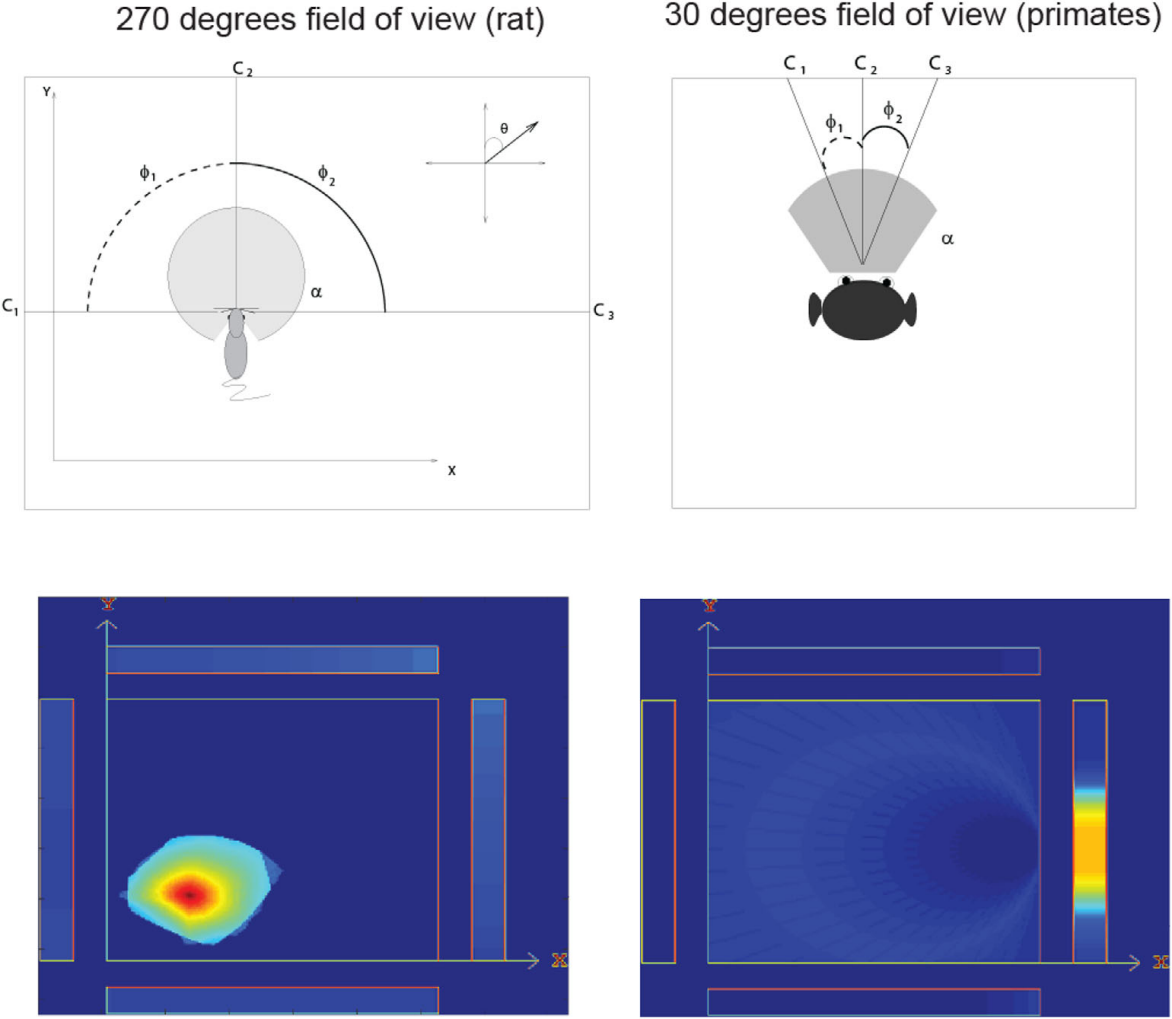
Simulation of rodent place cells (left) versus primate spatial view cells (right). The agent moved through a grid of all 200 × 200 places *x*,*y*. At each place, the head direction θ was rotated 5° increments. Hippocampal cells are activated by a set of three or more landmark visual cues within the field of view of the agent α. The firing rates of the hippocampal neurons depended on the angles φ subtended by the landmarks. The top left shows that for a rodent with a 270° field of view a combination of such cues defines a place. The top right shows that for a primate with a 30° field of view the combination of cues defines a spatial view. The sizes of the fields of view are shown by shading. The bottom left shows that in the simulations place fields arise with a 270° field of view, and the bottom right that spatial view fields arise on one of the walls indicated by the rectangles when the field of view is 30°. High firing rates are indicated by yellow‐red. (Details are provided in de Araujo et al. (2001).)

We start with the concept that in both primates and rodents, the dentate granule cells and the CA3 and CA1 neurons respond to combinations of their inputs. In primates the fovea provides high spatial resolution over a typical viewing angle of 5–10° in a complex natural scene as shown by the responses of macaque inferior temporal visual cortex (IT) neurons (with a mean receptive field size of 9°) (Aggelopoulos & Rolls, 2005; Rolls, Aggelopoulos, & Zheng, 2003). As a result, a combination of visual features in the spatial environment will produce a spatial view cell, the effective trigger for which will be a combination of visual features within a small part of space. This is illustrated in Figure 13 top right, where a primate hippocampal neuron responding to C_1_, C_2_, and C_3_ will effectively define a spatial view field. In rodents, in contrast, given the very wide visual field subtended by the retina, which may extend more than 270°, and the absence of a fovea, a combination of visual features learned over such a wide visual angle would define a position in space that is a place where the individual is located. This is illustrated in Figure 13 top left, where a rodent hippocampal neuron responding to C_1_, C_2_, and C_3_ with large angles between these cues will effectively define a place field where the individual is located.

The computational processes by which the hippocampal neurons would learn to respond to visual feature combinations in rats and primates could be similar, and include competitive learning in the dentate granule cells, autoassociation learning in CA3 cells, and competitive learning in CA1 cells (Rolls, 2016a; Rolls, 2021a; Treves & Rolls, 1994). Thus, the properties of primate spatial view cells and rodent place cells might arise by a similar computational learning process, but produce spatially different representations because primates are foveate and view a small part of the visual field at any one time, whereas rodents have a very wide visual field (see de Araujo et al. (2001)).

This was tested in a simulation in which the simulated animal explored its spatial environment, and hippocampal cells are activated by particular visual cues currently within the field of view, and learn by synaptic modification about these conjunctions of cues. If the field of view is 270°, then place cells were produced as a result of this exploration and learning, as shown in Figure 13. If the field of view was 30° in the simulations, then spatial view cells were produced as a result of this exploration and learning (Figure 13). Thus, the same computational learning process can lead to place cells with a large field of view as in rodents, and to spatial view cells in foveate animals such as primates including humans. This is relevant to the topic of this Special Issue of Hippocampus, in that it provides hypotheses about how viewed locations are involved in building different spatial representations in rodents and primates including humans. This is Rolls' hypothesis about how spatial view cells are formed in primates as a result of foveate vision (de Araujo et al., 2001).

There is a hint of something similar in rodents to what is produced by the fovea in primates, in that, when a rat is running along a linear track in which it can see primarily in one direction, then place cells can show more directional properties, in that they respond at a place when the rodent can see one view, but not if the rodent is in the same place and sees the other view (Acharya et al., 2016; McNaughton et al., 1983; Muller et al., 1994). It is predicted that if a rat's visual field was restricted, by, for example, a cone over the head, or in a virtual reality environment, then during learning new environments, or during development, the rat hippocampal place neurons might become more like macaque spatial view neurons.

This model thus shows that spatial representations may be produced by similar mechanisms in rodents and primates, but become different because of the primate fovea. Spatial view representations open up the issue of memory functions of the hippocampus involved in remembering where objects and rewards are in spatial scenes, an episodic memory function. The difference in spatial representations in the rodent and primate hippocampus does have implications for understanding how the hippocampus operates in spatial function and memory in primates including humans.

The actual implementation in the brain of the learning process to associate a combination of features, to produce a feature‐combination neuron, might include a short‐term memory trace in the associative synaptic learning rule that would make inputs that occur close together in time become associated together (Rolls, 2021f), in the same way as it is proposed helps to form invariant representations for object vision (Rolls, 2012; Rolls, 2016a; Rolls, 2021a; Rolls, 2021f). If different head directions occurred close together in time as in an open field, this might in rodents produce place cells with activity that was relatively invariant with respect to head direction. On the other hand, if the rodent was running in a straight arm of a maze, then the place cells would be predicted to respond primarily to the head direction in which the rodent was running, and the place cell shape would be predicted to be elongated in the direction of travel, and to reflect mainly what was seen in that direction of travel (cf. Derdikman et al., 2009). Boundary cells in rodents (Alexander et al., 2020; Lever et al., 2009) may similarly reflect the statistics of the spatial inputs when rodents reach a boundary and have to stop.

## 10 SIMILARITIES AND DIFFERENCES BETWEEN THE SPATIAL REPRESENTATIONS IN PRIMATES AND RODENTS

Despite the major differences in the spatial representations in the primate and rodent hippocampus, there are important similarities between the operation of the rodent and primate hippocampus, which indicate that the computational operation of these neural systems is comparable in rodents and primates, even though what is represented is different. Some of the similarities of the hippocampal system in primates and rodents were set out as follows (Rolls & Wirth, 2018).

First, the spatial representations are in both cases by most spatial view and place cells primarily allocentric. In monkeys, hippocampal spatial view cells during active locomotion in an open environment respond allocentrically to the view of a position in a spatial scene, relatively independently of the place where the monkey is in the open environment, of head direction, of eye position, and of where the spatial view field is relative to the monkey (Georges‐François et al., 1999; Rolls & O'Mara, 1995).

Second, the spatial representations can be updated idiothetically, by one's body or eye motion in primates, as in the rat.

Third, in both cases, the firing rates are low: in primates with a typical mean rate of 0.5 spikes/s, and a typical peak response rate of 17–20 spikes/s (Rolls et al., 1997b; Wirth et al., 2017). This matches numerous accounts of firing properties in rat hippocampus.

Fourth, spatial view cells may fire just before the eyes reach the center of the spatial view field, and may have their maximal response soon after the eyes reach the spatial view field, and decrease somewhat after that, i.e., show some adaptation (Wirth et al., 2017). Analogous findings have been described for rodent hippocampal cells which generate spike sequences lasting about 2 s as rats traverse a place field. The firing rate in the place field shows an asymmetry which changes with experience: as rats become familiar with an environment; cells show an increase in rate before animals reach the place field, followed by a gradual decrease as rats leave the field (Mehta et al., 2000).

Fifth, in macaques, there is evidence for independent representation about spatial view by hippocampal neurons, in that the information rises linearly with the number of neurons (Rolls et al., 1998). This independence arises when the response profiles of the neurons are uncorrelated (Rolls, 2021a; Rolls & Treves, 2011). This is a powerful encoding, because the number of stimuli (e.g., spatial views) rises exponentially with the number of neurons. (Of course, this independence applies only in a high‐dimensional environment, and saturates to the limit in lower dimensional environments (Rolls, 2016a; Rolls, 2021a; Rolls & Treves, 2011).) Ensemble encoding by populations of neurons is found in rodents (Wilson & McNaughton, 1993), and it would be interesting to know whether the coding by different neurons is also independent in rodents.

Sixth, rodent place cells may respond differently on the trajectory to approach a goal depending on the state of the animal (Ferbinteanu et al., 2011; Fyhn et al., 2002; Wood et al., 2000), as in primates (Wirth et al., 2017). This implies that place cells support cognition in both species.

Seventh, in macaques, object‐spatial view neurons are found (Rolls et al., 2005), and one‐trial object‐place learning and recall can occur (Rolls & Xiang, 2006). In rodents, object‐place or odor‐place neurons have been described (Kim et al., 2011; Komorowski et al., 2009). The presence of a barrier or boundary, which might be thought of as an object, in a place, may also be encoded by rodent hippocampal (Rivard et al., 2004; Wang, Chen, & Knierim, 2020; Wang, Monaco, & Knierim, 2020) and retrosplenial (Alexander et al., 2020) neurons.

Eighth, in macaques, reward‐spatial view neurons are found (Rolls & Xiang, 2005), and cells are found to encode reward outcomes (Brincat & Miller, 2015; Wirth et al., 2009). In rodents reward‐place neurons have been described (Tabuchi et al., 2003), and it was found that place cells are more active after the receipt of a reward (Singer & Frank, 2009).

Ninth, in rodents, distal room cues can influence place cells (Acharya et al., 2016; Aronov & Tank, 2014; Knierim & Rao, 2003; Shapiro et al., 1997). However, this is different to the encoding of a location in a scene that is provided by primate spatial view cells, in that in rodents the distal room cues are used to encode the place where the rodent is located.

Tenth, in both primates and rodents, restricting the view of the environment may have analogous effects. In primates navigating through spatial trajectories in a star maze, many neurons had their responses influenced by place, the direction in which the macaque was facing, and by the part of the trajectory being performed (Wirth et al., 2017). In rats tested in an open foraging environment in which all places and head directions occur, rat place cells tend to have only small directional selectivity. However, in rats tested in linear runways in which a task may be performed and in which only some combinations of head direction and place are common, place cells may be quite directional (Acharya et al., 2016). A possibility is that in the foraging situation used by Rolls et al. (Georges‐François et al., 1999; Robertson et al., 1998; Rolls et al., 1997b; Rolls et al., 1998), all places, views, and head directions occurred, and the cells were dominated by where the animal looked, and not by place or head direction. In contrast, if the macaque in a VR environment was constrained by the star maze to visit only certain places with particular spatial views and head directions that were frequently viewed from each of those places, and was performing a task that required a trajectory to a goal, then the neurons might reflect not only where the macaque was looking in the environment, but also the place from which the looking occurred, and so forth (Wirth et al., 2017). That is, if certain combinations of spatial view and place are common in an environment, then hippocampal neurons would be likely to encode primarily the combinations of spatial views and the places from which they are primarily seen.

Eleventh, whole body motion cells which respond to either linear velocity or angular velocity are present in the macaque hippocampus (O'Mara et al., 1994), and have more recently been described as speed cells in the rodent entorhinal cortex (Kropff et al., 2015).

Twelfth, macaques (Robertson et al., 1999; Rolls, 2005), as well as rodents (Taube et al., 1990; Taube et al., 1996), have head direction cells in the presubiculum/subiculum.

These considerable similarities between the responses of neurons found in the rodent and primate hippocampal system provide evidence that the systems operate in similar ways in primates and rodents, but with different spatial representations (Rolls & Wirth, 2018). The different representations can be related to the evolution of the primate fovea, and its effects on object representations in the ventral cortical visual stream, and on systems in the primate dorsal visual stream for eye movements to produce foveation and for an interface to produce visually guided actions in the connected parietal cortical areas (Rolls, Deco, et al., 2022b; Rolls, Deco, et al., 2022c). Moreover, foveation of an object in primates is an efficient way to transmit the coordinates from a visually fixated object to the dorsal visuomotor system (Rolls, Aggelopoulos, & Zheng, 2003; Rolls & Deco, 2002) in the parietal cortex (Andersen & Cui, 2009; Galletti & Fattori, 2018; Gamberini et al., 2020; Rolls, Deco, et al., 2022c). Further, the saccadic system of primates (including humans) enables a primate in one place to look toward one part of a scene and recall the object there, and then to saccade to another point in the scene and recall the object there. There is no evidence for anything similar in rodents, and this highlights an important difference between primate and rodent hippocampal spatial representation and memory systems that arises because of the primate fovea.

## 11 HIPPOCAMPAL COMPUTATIONAL SIMILARITIES AND DIFFERENCES FOR VIEW BETWEEN PRIMATES AND RODENTS

Although the spatial representations in the primate and rodent hippocampus are different, it is proposed that the underlying computations performed are similar (Rolls & Wirth, 2018).

A quantitative and detailed theory and model of how the hippocampus operates as a memory system, and of the way in which information stored in the hippocampus could be recalled back to the neocortex, has been developed (Kesner & Rolls, 2015; Rolls, 1989b; Rolls, 2016a; Rolls, 2018a; Rolls, 2021a; Treves & Rolls, 1992; Treves & Rolls, 1994), with the architecture illustrated in Figure 10. In the theory, the CA3 network forms an autoassociative or attractor memory, given the associatively modifiable recurrent connectivity between CA3 neurons. According to this theory, this system operates similarly in rodents and primates, to allow arbitrary associations between places in rodents, or spatial views in primates, and objects or rewards, to be rapidly formed, and later the whole memory to be recalled from a part. For example, the location of an object might be recalled in CA3 when an object recall cue was presented.

Temporal sequences for episodic memory may be remembered by replacing the location cells with the timing cells described by Eichenbaum and colleagues (Eichenbaum, 2014; Howard et al., 2014; Howard & Eichenbaum, 2015; Kraus et al., 2013; Kraus et al., 2015; Macdonald et al., 2011), and this applies to primates (Naya & Suzuki, 2009) including humans (Umbach et al., 2020) too. A theory of the generation of hippocampal time cells (Rolls & Mills, 2019) from entorhinal cortex cells with time courses of their firing changing over tens or hundreds of s (Tsao et al., 2018) could apply equally to primates as rodents. Indeed, consistent with this, neurons in the monkey entorhinal cortex have a spectrum of time constants of their firing (Bright et al., 2020). The theory of the operation of time cells shows a mechanism by which forward and reverse replay of memories could be produced (Rolls & Mills, 2019), and rather than these phenomena being involved in memory consolidation, it is proposed that at least in humans the reward value of episodic memories helps to influence their recall and whether therefore they are retrieved in the neocortex and reorganized for semantic storage and consolidation in the neocortex (Rolls, 2022). The temporal order of events in an episodic memory might also be implemented using a temporal asymmetry of the synaptic modification in, for example, the CA3 recurrent collaterals, but such models are expensive in terms of memory capacity and can probably only be used for short sequences in the order of 2 s (Akrami et al., 2012; Hasselmo et al., 2010; Sompolinsky & Kanter, 1986; Spalla et al., 2021).

The leading factor in the number of memories that can be stored and successfully recalled in this system is the number of synapses onto any one CA3 neuron by the associatively modifiable synapses from the recurrent collaterals of other CA3 neurons. With sparse representations, the number of memories that can be stored is in the order of the number of synapses onto each CA3 neuron (Treves & Rolls, 1991). It is interesting that an important difference in evolution arises in humans, in which the CA3 neurons are not well connected across the midline by the hippocampal commissure, given what is found in macaques (Amaral et al., 1984). In rodents, the CA3‐CA3 in the two hippocampi are as much connected as within the hippocampus on one side in the brain, and this enables the rodent CA3 hippocampal network to operate as a single hippocampus (Rolls, 2016a; Rolls, 2021a). In humans, there appear to be effectively separate left and right CA3 hippocampal networks given the poor commissural connectivity. Consistent with this point, there is evidence that the right human hippocampus specializes in spatial including object‐place and reward‐place memories, and the left hippocampus specializes in more language/word‐related memory processes (Barkas et al., 2010; Bonelli et al., 2010; Burgess et al., 2002; Crane & Milner, 2005; Sidhu et al., 2013). The adaptation here is that humans have twice the memory capacity of a hippocampal system connected across the midline as in rodents; and that associations are not typically made between words and their position in space, for the latter are not part of what is implemented for human language. The implication for spatial view neurons in humans is that they may be found more in the right hippocampus and possibly more in the right PSA.

Key points made here are that the primate parietal cortex may implement idiothetic update of hippocampal and parahippocampal spatial view neurons; and that recall of spatial view information from the hippocampus to neocortical areas such as the parietal cortex may be involved in navigation and in visuomotor processing to reach for and grasp objects or rewards at recalled locations in the world. A possible implication is that the primate including human hippocampus may be especially involved in episodic memory, but that the actual computations for navigation and movements in the environment for navigation and visuomotor function may be implemented outside the hippocampus, in neocortical areas such as the parietal cortex. Consistent with this hypothesis, lesions of the human neocortex can produce topographical agnosia and inability to navigate (Barton, 2011; Kolb & Whishaw, 2015), and the retrosplenial cortex is implicated in navigation (Alexander & Nitz, 2015; Byrne et al., 2007; Epstein, 2008; Vann et al., 2009; Vedder et al., 2017). In more detail, lesions restricted to the hippocampus in humans result only in slight navigation impairments in familiar environments, but rather strongly impair learning or imagining new trajectories (Bohbot & Corkin, 2007; Clark & Maguire, 2016; Maguire et al., 2016; Spiers & Maguire, 2006; Teng & Squire, 1999). In contrast, lesions in regions such as the parietal cortex or the retrosplenial cortex produce strong topographical disorientation in both familiar and new environments (Aguirre & D'Esposito, 1999; Habib & Sirigu, 1987; Kim et al., 2015; Maguire, 2001; Takahashi et al., 1997). This suggests that the core navigation processes (which may include transformations from allocentric representations to egocentric motor commands) is performed independently by neocortical areas outside the hippocampus, which may utilize hippocampal information related to recent memories (Ekstrom et al., 2014; Miller et al., 2013).

Further, and consistent with the spatial view cells found in nonhuman primates, regions of the human hippocampal formation can become activated when people look at spatial views (Epstein & Kanwisher, 1998; O'Keefe et al., 1998). Moreover, the right human hippocampus is activated during mental navigation in recently learned but not in highly familiar environments (Hirshhorn et al., 2012). Mental navigation in familiar environments produces activation of cortical areas such as the lateral temporal cortex, posterior parahippocampal cortex, lingual gyrus, and precuneus (Hirshhorn et al., 2012). Further, as noted above, patients with anterograde amnesia may not be impaired in navigation in familiar environments, as contrasted with new environments (Clark & Maguire, 2016; Maguire et al., 2016). The implication is that, at least in primates, the hippocampus may be involved in episodic memory, and that neocortical regions implement navigation (helped when it is useful by recent memories recalled from the hippocampus).

In contrast, the view has often been held that the rodent hippocampus implements navigation. Indeed, in rodents, the existence of place cells has led to hypotheses that the rodent hippocampus provides a spatial cognitive map, and can implement spatial computations to perform navigation. These navigational hypotheses could not account for what is found in the primate hippocampus. An alternative that is suggested is that, in both rodents and primates, hippocampal neurons provide a representation of space (which for rodents is the place where the rat is located, and for primates includes positions “out there” in space), which are used as part of an episodic memory system. In primates, this would enable formation of a memory of where an object was seen (Rolls, 1987; Rolls, 1989b; Rolls, 2016a; Rolls, 2018a; Rolls, 2021a; Rolls & Kesner, 2006). In rodents, this would enable the formation of memories of where particular objects (defined by olfactory, tactile, and taste inputs for instance) were found (Kesner & Rolls, 2015). Consistent with this theory of hippocampal function, one‐trial object‐place memory in rodents requires the hippocampus (Day et al., 2003; Kesner & Rolls, 2015; Takeuchi et al., 2014); texture sensed by whiskers and the places of rewards are reflected in neuronal firing (Itskov et al., 2011); some hippocampal neurons respond to behavioral, perceptual, or cognitive events, independently of the place where these events occurred, and may thus be useful for memory functions (Komorowski et al., 2009; Wood et al., 1999; Wood et al., 2000); hippocampal neurons may be activated following relocation of a target object to a new place (Fyhn et al., 2002); some hippocampal neurons alter their response when a different recording chamber is placed in the same location in the room (Leutgeb et al., 2005); and another continuous dimension than place, namely auditory frequency, can be mapped by rodent hippocampal neurons (Aronov et al., 2017). Thus, in primates, and probably also in rodents, the hippocampal representation of space may be appropriate for the formation of memories of episodic events (for which there is typically a spatial component). These memories would be of use in spatial navigation.

## 12 CONCLUDING POINTS, AND FUTURE RESEARCH

The research described here shows that primates including humans have allocentric spatial view cells in the hippocampus and parahippocampal cortex, and that these are implicated in episodic memory, and in navigation at least when it is guided by memory recall. The recent investigations of the connectivity of the human hippocampal system with other cortical areas emphasize important issues: The connectivity with the orbitofrontal and anterior cingulate cortex supports the hypothesis that reward is not only an important component of episodic memory, but also that reward (acting in part via cholinergic pathways) may influence what memories are consolidated (Rolls, 2022; Rolls, Deco, et al., 2022e). That connectivity also emphasizes the point that the reward pathways to the hippocampus and other regions provide the goals for navigation (Rolls, 2022; Rolls, Deco, et al., 2022e). The connectivity of the hippocampal system with the parahippocampal gyrus TH and ventromedial visual areas (VMV1‐3) (Ma et al., 2022; Rolls, Deco, et al., 2022d; Rolls, Wirth, et al., 2022) leads to the hypothesis that the “where” scene representation is in fact of ventral visual stream origin. The connectivity of the hippocampal system with the parietal cortex (Rolls, Deco, et al., 2022c; Rolls, Deco, et al., 2022d; Rolls, Wirth, et al., 2022) leads to the hypotheses that the parietal areas may implement idiothetic update of spatial view cells; and in addition may implement most of the computations involved in human navigation and visuomotor reaching and grasping, with the hippocampus providing the episodic memory that may guide such navigation and action. It is thus proposed that there are two “where” systems in primates including humans. The presence of a fovea in primates, and the highly developed temporal lobe for invariant visual object recognition and for the representation of scenes in the parahippocampal gyrus TH enables memory and navigation that can be based on high visual acuity representations of distant spatial scenes. The presence of foveate vision in primates has also led to the need for parietal cortex systems to be involved in eye movement control, and in the idiothetic update that includes compensation for self‐movement including eye movements when representing spatial locations “out there” when the view details are obscured. This provides the basis for navigation and episodic memory in primates including humans that can utilize distant landmarks in scenes. This contrasts with the situation on rodents, in which the visual system may provide a much less clear view of distant locations in scenes, so that navigation and episodic memory may rely more on proximal cues and path integration from place to place.

New discoveries made in the research in primates including humans described here that are different from and not predicted from investigations in rodents (Bicanski & Burgess, 2018; Burgess & O'Keefe, 1996; Hartley et al., 2014; McNaughton et al., 1983; O'Keefe, 1979; O'Keefe et al., 1998; O'Keefe & Krupic, 2021) include the following:

1. Many hippocampal neurons in primates including humans encode locations in viewed spatial scenes, not the place where the individual is located. This fits with the great development of the primate visual system, with the primate fovea, and with the use of eye movements to fixate different parts of a scene, or objects. It also fits with the activation reported to viewed visual scenes in the human PSA.
2. Primate hippocampal spatial view neurons that encode “where” information receive from *ventral* visual stream regions (rather than the parietal cortex), and encode visual features present in scenes and their location by associating together spatial features based on their nearness in space and hence the probability that they are co‐active in their firing to implement the learning in a continuous attractor network in the PSA.
3. In primates, the parietal cortex is involved in the coordinate transforms that are needed for path integration including from retinal to head‐based coordinates by taking into account eye position, as well as from head‐based to at least compass direction‐based world allocentric coordinates. These coordinate transforms performed in the parietal cortex then via demonstrated connectivity to the PSA are likely to update parahippocampal spatial view neurons, with idiothetic update being one of their properties that we discovered been discovered. In contrast, in rodents, path integration typically is described as involving update of place cells by head direction and distance traveled, and as taking place in brain regions such as the hippocampus or a retrosplenial region.
4. Navigation in primates including humans is often from viewed landmark to viewed landmark as can be implemented by spatial view cells, and for familiar environments may be performed with neocortical regions rather than by the hippocampus. In contrast, navigation as implemented in rodents using place cells, head direction, and distance traveled is much less powerful, as is easily demonstrated in humans who try to navigate over a route with their eyes closed.
5. The primate hippocampus receives view invariant representations of objects that can be associated with the viewed location in a scene signaled by spatial view cells, to enable the formation of episodic memories. In contrast, in rodents view invariant representations of single fixated objects in a scene are not a feature of the rodent visual system, and it is not clear how the visual object‐visual location capability of human episodic memory is implemented in rodents, and indeed it has been argued even recently that hippocampal representations in rodents are primarily about places (O'Keefe & Krupic, 2021). However, associations of place with stimuli present at localized places such as an odor or a somatosensory texture cue can probably be utilized in a place‐specific form of episodic memory in rodents (Kesner & Rolls, 2015 ; Rolls & Kesner, 2006).
6. In addition, reward information reaches the primate (Rolls & Xiang, 2005) including human hippocampus from the orbitofrontal and pregenual anterior cingulate cortex (Rolls, Deco, et al., 2022e), and in the hippocampus, this can play an important role in episodic memory of which part is reward value, and can also provide the goals for navigation (Rolls, 2022). Further, the highly developed human orbitofrontal cortex (Rolls, 2019a; Rolls, 2019b) has effective connectivity to the basal forebrain cholinergic neurons that connect to the neocortex, and the pregenual anterior cingulate to the cholinergic septal nuclei (Figure 9) (Rolls, Deco, et al., 2022e), which provide for the orbitofrontal cortex reward value system to play a key role in memory consolidation in humans (Rolls, 2022). In contrast, in rodents memory consolidation is described as involving sleep and hippocampal replay of memories (Foster, 2017; Skelin et al., 2019 ; Wilson & McNaughton, 1994).

The research described here suggests many areas for future research. One is how view computations may also be important in the hippocampal system of rodents and other species, which other papers in this Special Issue of Hippocampus address. It could be interesting to explore spatial representations in rodents when the field of view is restricted to a small cone to emulate a primate fovea; and to explore how navigation operates in primates including humans when only a low acuity representation of the world is provided by convolving the visual input with the visual spatial transfer function of a rodent such as a rat (i.e., the visual acuity as a function of eccentricity).

Another issue is whether spatial view neurons of the type described here in the macaque hippocampus and parahippocampal gyrus are found in the human PSA and related regions as proposed here.

Another point emphasized for future research is that to dissociate spatial view from head direction or facing direction, and from place, and from egocentric location and retinal and eye position encoding, it is essential to record eye position and head direction as well as the place of the individual, and to ensure adequate sampling of all spatial views from all places and with all head directions and eye positions and scene facing directions.

Another key point for future research raised by the connectivity in humans described here is on the role of the vmPFC, orbitofrontal cortex, and anterior cingulate cortex in memory. It is proposed here that the orbitofrontal cortex and vmPFC do not perform spatial computations, but influence them and memory by introducing reward inputs to the hippocampus which are key parts of episodic memory and navigation, and also by influencing the cholinergic system in the septal region that projects to the hippocampus, and in the basal forebrain that projects to the neocortex (Rolls, 2022; Rolls, Deco, et al., 2022e), and which are implicated in the synaptic mechanisms involved in memory consolidation (Giocomo & Hasselmo, 2007; Hasselmo, 1999; Hasselmo & Bower, 1993; Hasselmo & Giocomo, 2006; Hasselmo & McGaughy, 2004; Hasselmo & Sarter, 2011; Newman et al., 2012).

## CONFLICT OF INTEREST

The author declares no conflict of interest.

## Supporting information


**APPENDIX S1** Supporting Information

## Data Availability

Videos that illustrate the responses of macaque hippocampal spatial view cells are provided in the Supplementary Material. One example of a spatial view cell is in file az033.mp4, which illustrates a small part of the data from this neuron that was included in the analysis of the coordinate system used by spatial view neurons (Georges‐François et al., 1999). The enclosure is the central square, the four walls are the rectangles surrounding the square with the height on the wall indicated by the distance in the wall rectangle away from the center of the diagram, and a red dot is added to this wall plot whenever the cell fires an action potential. The position and head direction of the macaque are indicated by the triangle, and the eye gaze direction by the line projected to the edge of the enclosure, which is black when the cell is not firing, and red when the cell fires. The firing of other spatial view cells during active locomotion are illustrated in Supplementary Material files: av232.mp4, and av191.mp4. Programs written in MATLAB (which also run under the freeware Octave) to illustrate the operation of autoassociation (attractor) and related networks are available in connection with *Cerebral Cortex: Principles of Operation* (Rolls, 2016a) and *Brain Computations: What and How* (Rolls, 2021a) at https://www.oxcns.org/NeuronalNetworkSimulationSoftware.html with Appendices explaining their operation available at https://www.oxcns.org/papers/Cerebral Cortex Rolls 2016 ContentsandAppendices.pdf.
